# Gut bacteria of weevils developing on plant roots under extreme desert conditions

**DOI:** 10.1186/s12866-019-1690-5

**Published:** 2019-12-30

**Authors:** Fengqun Meng, Nitsan Bar-Shmuel, Reut Shavit, Adi Behar, Michal Segoli

**Affiliations:** 1 Mitrani Department of Desert Ecology, The Swiss Institute for Dryland Environmental and Energy Research, The Jacob Blaustein Institutes for Desert Research, Ben-Gurion University of the Negev, Midreshet Ben-Gurion, Israel; 2 French Associates Institute for Agriculture and Biotechnology of Drylands, The Jacob Blaustein Institutes for Desert Research, Ben-Gurion University of the Negev, Midreshet Ben-Gurion, Israel; 3 Division of Parasitology, Kimron Veterinary Institute, Bet Dagan, Israel

**Keywords:** Desert ecosystem, Symbiont, Beetle, Weevil, Nutrient, *Citrobacter*

## Abstract

**Background:**

Many phytophagous insects, whose diet is generally nitrogen-poor, rely on gut bacteria to compensate for nutritional deficits. Accordingly, we hypothesized that insects in desert environments may evolve associations with gut bacteria to adapt to the extremely low nutrient availability. For this, we conducted a systematic survey of bacterial communities in the guts of weevils developing inside mud chambers affixed to plant roots in the Negev Desert of Israel, based on 16S rRNA gene amplicon sequencing.

**Results:**

Our analyses revealed that gut bacterial communities in weevil larvae were similar across a wide geographical range, but differed significantly from those of the mud chambers and of the surrounding soils. Nevertheless, a high proportion of bacteria (including all of the core bacteria) found in the weevils were also detected in the mud chambers and soils at low relative abundances. The genus *Citrobacter* (of the Enterobacteriaceae family) was the predominant group in the guts of all individual weevils. The relative abundance of *Citrobacter* significantly decreased at the pupal and adult stages, while bacterial diversity increased. A mini literature survey revealed that members of the genus *Citrobacter* are associated with nitrogen fixation, recycling of uric acid nitrogen, and cellulose degradation in different insects.

**Conclusions:**

The results suggest that although weevils could potentially acquire their gut bacteria from the soil, weevil host internal factors, rather than external environmental factors, were more important in shaping their gut bacterial communities, and suggest a major role for *Citrobacter* in weevil nutrition in this challenging environment. This study highlights the potential involvement of gut bacteria in the adaptation of insects to nutritional deficiencies under extreme desert conditions.

## Background

Many phytophagous insects, whose diet is often nutritionally suboptimal and nitrogen-poor, have been shown to rely on gut bacteria to complement their nutritional requirements [1–3]. For example, mutualistic interactions with diazotrophic bacteria (i.e.*,* that fix atmospheric nitrogen) were reported in several wood-eating termite species [4, 5], a wood-eating cockroach [6], several bark and stag beetle species [7–9] [10], and fruit flies [11]. Associations with uricolytic bacteria that recycle uric acid nitrogen, which is later re-incorporated into insect tissues, has been reported in a wood-eating termite [12], a drupe-eating bug [13], fruit flies [11], and several herbivorous ants [14]. Some phytophagous insects also rely on their gut bacteria for the synthesis of essential molecules such as particular amino acids and vitamins. For example, plant sap-feeders (e.g.*,* many hymenopterans), are often associated with obligate gut bacteria, such as *Ishikawaella* that supply them with essential amino acids [15, 16]. In addition, a large group of phytophagous insects feeding on bark or wood, which are rich in fastidious polymers, harbor gut bacteria responsible for the breakdown of ingested polymers into simpler forms that can be directly assimilated by the host insect [17]. In accordance, wood-eating insects were shown to harbor gut bacteria that aid them with cellulose digestion, while these bacteria are conspicuously absent from foliage and detritus-feeders [18].

Knowledge of insect gut bacteria has been primarily developed from insect pests in forest and agricultural ecosystems in an effort to promote pest control strategies (e.g.*,* see reviews [2, 19]), while it is less well characterized in other environments. In desert ecosystems, soil nutrient availability, and particularly nitrogen, is considered to be limited because of low soil moisture coupled with high temperatures and high soil salinity [20, 21], most likely reducing nutrient uptake and nitrogen concentrations in desert plants [22]. Therefore, phytophagous insects in desert environments may experience extreme nitrogen limitation, which may lead them to evolve associations with specific gut bacteria as an adaptation to the exceptionally low nutrient availability.

**Fig. 1:**
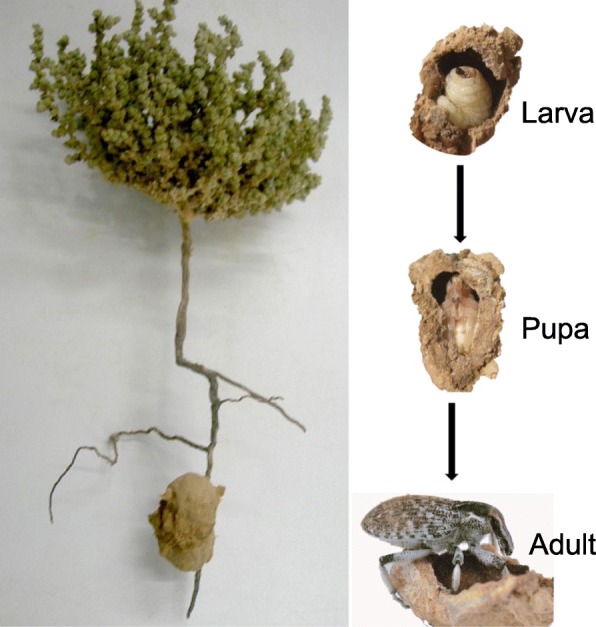
Mud chamber affixed to the root of *Salsola inermis*, and larva, pupa, and adult (*Menecleonus virgatus*) in the mud chamber

Here, we took a first step in addressing this hypothesis by focusing on the gut bacteria of weevils (*Conorhynchus palumbus* Olivier and *Menecleonus virgatus* Schoenherr; Coleoptera: Curculionidae: Lixinae) that develop singly in a mud chamber affixed to the roots of two summer annual plants of the genus *Salsola* (*Salsola inermis* Forssk and *S. incanescens* Mey; Chenopodiaceae) [23] (Fig. 1). These weevils are widely distributed in the Negev Desert of Israel [24]. The weevil completes most of its life cycle during the summer within a mud chamber underground, where the larva presumably feeds on the plant fluids [25]. The adults emerge during the spring and are active leaf chewers. *S. inermis* and *S. incanescens* plants grow in salty desert loessial soils, which are low in nutrient availability (e.g.*,* with a relative nitrogen content of ~ 0.02% in the soil [25]); thus, these plants are also poor in nutrient content (e.g.*,* with a relative nitrogen content of ~ 0.66% in *S. inermis* [25], versus a global average of 1.58% in plant leaves [26]). Hence, it is unclear how these weevils obtain sufficient nutrients for their development.

Many weevils have been shown to harbor maternally transmitted intracellular bacteria (e.g.*,* bacteriome-localized *Nardonella, Sodalis* and *Curculioniphilus*) that synthesize and provide essential amino acids to promote weevil development [27–30]. In addition, gut bacteria of various weevils have been reported to be involved in polymer degradative activities [8, 31–35], e.g.*,* diterpene-degrading bacteria in conifer-feeding weevils [36, 37]. Nitrogen-fixing and nitrogen-recycling bacteria have also been suggested to supplement their host’s diet in phloem-feeding weevils [8, 9].

In this study, to investigate the potential role of gut bacteria of desert weevils, we conducted 16S rRNA gene amplicon sequencing of bacterial communities in the guts of these weevils throughout their life cycle and at different geographical locations. We also investigated bacterial communities in surrounding soils and in the mud chamber itself to examine to what extent the external soil environment vs. the internal physiological environment inside the weevil guts shapes the composition of their gut bacterial communities. Finally, we tested whether weevil gut communities are dominated by certain bacteria, as the high dominancy and stability of certain bacteria are often linked with their functional importance to their insect hosts [36, 38]. To this end, we conducted a literature survey to learn about the potential biological functions of the most dominant bacteria within the weevils.

## Results

### Bacterial community composition in guts of *C. palumbus* weevils, mud chambers, and the surrounding soils

**Fig. 2:**
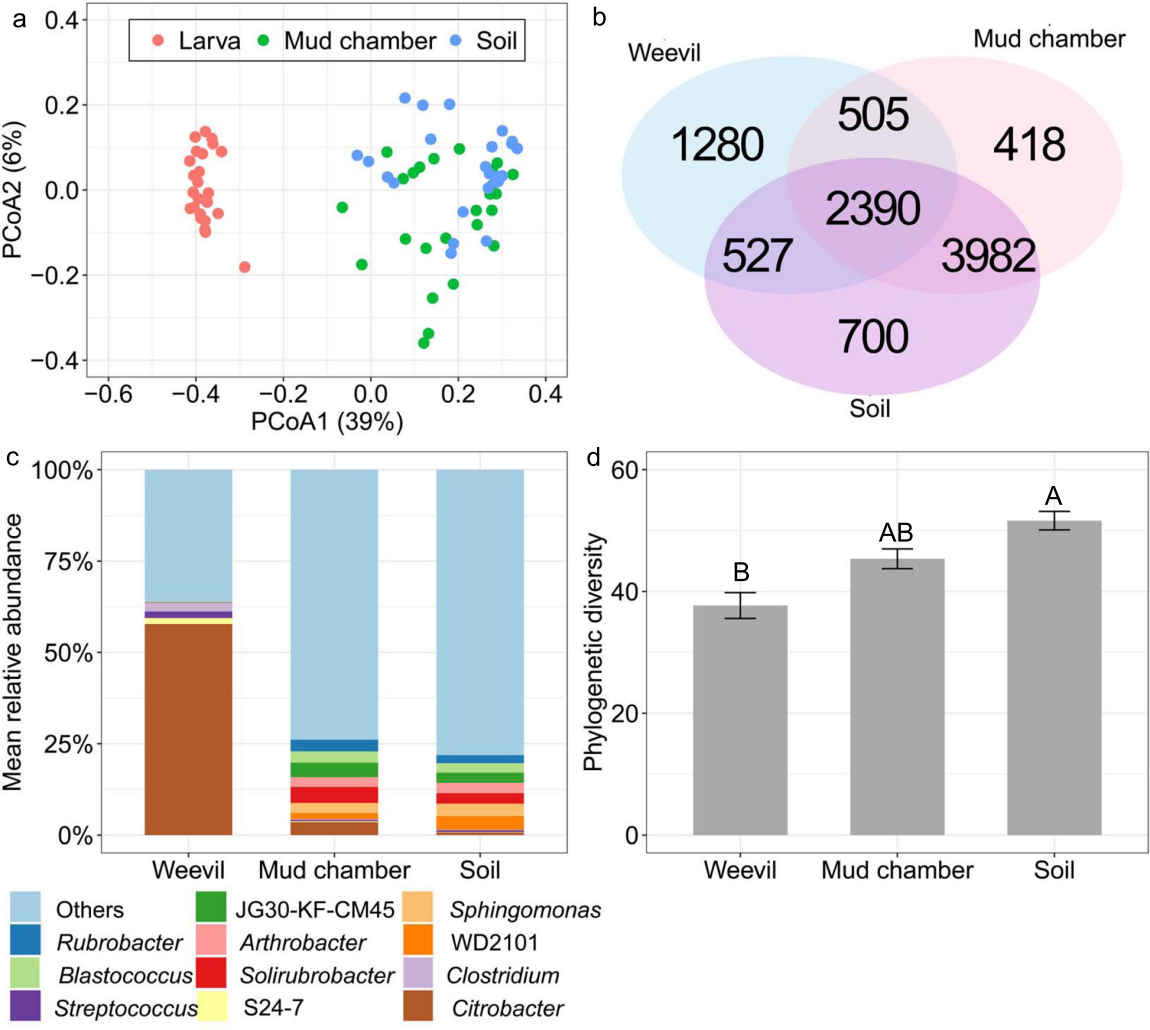
Bacterial community composition in the guts of *Conorhynchus palumbus* (*N* = 25), mud chamber (*N* = 25) and surrounding soil (*N* = 25). (**a**) PCoA plot displaying unweighted UniFrac distance. The percent variation explained by each principle coordinate is shown. (**b**) Venn diagram representing the number of OTUs that are unique to each of the sample type and shared between them. (**c**) Mean relative abundance of bacterial genera. For weevil, only those genera with > 1% mean relative abundance across all weevil samples are shown; for mud chamber and soil, only the top five genera are shown, whereas all remaining sequences are represented as others. S24–7, Actinomycetales, Rhodobacteraceae, and Frankineae are provided because the phylotypes were not classified to lower taxonomic levels. (**d**) Phylogenetic diversity. Columns with different letters are different at *P* < 0.05 based on Tukey’s post hoc tests

Permutational multivariate ANOVA (PERMANOVA) and principal coordinates analysis (PCoA) based on unweighted UniFrac distance showed that weevils, mud chambers, and soils significantly differed in their bacterial communities (PERMANOVA: *F*_*2,74*_ = 21.73, *R*^*2*^ = 0.38, *P* = 0.001; Fig. 2a). Post hoc pairwise tests showed that bacterial communities from the weevil guts were significantly different from those of the mud chambers (*P* = 0.003) and of the surrounding soils (*P* = 0.003); and that those of the mud chambers were also significantly different from those of the surrounding soils (*P* = 0.009), although PCoA plots did not indicate a clear segregation between them. Results of tests based on binary Jaccard index, abundance Jaccard index and weighted UniFrac distance were qualitatively similar (see Additional file 1: Appendix A).

Consistently, as shown in the Venn diagram, the proportion of shared operational taxonomic unit (OTU) between soil and mud chamber (84%) was higher than the corresponding proportion of shared OTUs between weevil and mud chamber (61%) and between weevil and soil (62%) (Fig. 2b). The proportions of unique OTUs in weevils, mud chambers, and soils were 27, 17, and 29%, respectively, mainly corresponding to rare OTUs (relative abundance below 0.1%). All the 66 weevil core OTUs (defined as those that were present in at least 70% of all weevil samples) were observed in both mud chamber and surrounding soil but at a relatively low abundance and accounted for a total of 74 ± 3% (SE) of the relative abundances in weevil guts.

The differences in bacterial communities were evident also at the genus level (PERMANOVA: *F*_*2,74*_ = 43.03, *R*^*2*^ = 0.56, *P* = 0.001; Fig. 2c). The weevil gut bacteria were dominated by *Citrobacter*, followed by *Clostridium*, *Streptococcus*, and S24–7 (the Bacteroidetes family); while several members of Lachnospiraceae, Ruminococcaceae and Anaerolineaceae, were unique to it. The predominancy of the genus *Citrobacter* in weevils was mainly driven by the high relative abundance of a single OTU (though the analysis showed 28 OTUs binned to *Citrobacter*), which was also present but less abundant in the mud chambers and surrounding soils (mean relative abundances of 42.85 ± 3.13%, 2.87 ± 1.04%, and 0.53 ± 0.30%, respectively). The five most abundant bacteria in the mud chambers were *Solirubrobacter*, JG30-KF-CM45 (the Thermomicrobia order), *Citrobacter*, *Rubrobacter*, and *Blastococcus*; while *Winogradskyella*, *Sulfitobacter* and *Actinokineospora* were unique to it. The five most abundant bacteria in surrounding soils were WD2101 (the Phycisphaerae order), *Sphingomonas*, *Solirubrobacter*, *Arthrobacter*, and JG30-KF-CM45; while Halotalea, *Crinalium*, *Niastella, Sediminibacter* and several members of the Cyanobacteria phylum were unique to it. The soil bacterial community was qualitatively similar to what was found in a previous study in the same region of the Negev Desert [39] (Additional file 1: Appendix B).

A similarity percentage analysis (simper) revealed 12 bacteria contributing to 50% of the dissimilarity between the weevil gut and the mud chamber, including *Citrobacter* (contributing to 32% of the dissimilarity) being more abundant in the weevil guts, and the other 11 bacteria being more abundant in the mud chambers (Table 1). A similarity percentage analysis between the weevil gut and the surrounding soil showed that 13 bacteria contributed to 50% of the dissimilarity, including *Citrobacter* (contributing to 33% of the dissimilarity) and *Clostridium* being more abundant in the weevil gut, and the other 11 bacteria being more abundant in the surrounding soil (Table 1).

**Table 1:** Summary of bacteria that contributed to 50% of the dissimilarity between the weevil gut and the mud chamber, and between the weevil gut and the surrounding soil, revealed by similarity percentage analyses. + represents more abundant, − represents less abundant

Weevil gut vs. Mud chamber				Weevil gut vs. Soil			
Bacteria	%Contribution	Weevil	Mud chamber	Bacteria	%Contribution	Weevil	Soil
*Citrobacter*	31.79	+	–	*Citrobacter*	32.75	+	–
*Solirubrobacter*	2.51	–	+	*Clostridium*	1.31	+	–
JG30-KF-CM45	2.29	–	+	*Solirubrobacter*	1.62	–	+
*Rubrobacter*	1.85	–	+	JG30-KF-CM45	1.57	–	+
*Blastococcus*	1.77	–	+	*Rubrobacter*	1.23	–	+
*Conexibacter*	1.75	–	+	*Blastococcus*	1.5	–	+
*Sphingomonas*	1.62	–	+	*Conexibacter*	1.21	–	+
*Arthrobacter*	1.55	–	+	*Sphingomonas*	1.95	–	+
*Saccharopolyspora*	1.44	–	+	*Arthrobacter*	1.62	–	+
Actinomycetales	1.44	–	+	WD2101	2.1	–	+
*Chloroflexi*	1.37	–	+	Rhodobacteraceae	1.49	–	+
AKIW543	1.33	–	+	*Gemmatimonas*	1.32	–	+
				Frankineae	1.12	–	+

Weevil gut bacteria had the lowest alpha diversity based on the phylogenetic diversity, and there was no significant difference between the mud chamber and the surrounding soil (Generalized linear mixed model (GLMM): *F*_*2,74*_ = 6.45, *P* = 0.003; Fig. 2d). Similar results were obtained when comparing the Chao1 richness index, Shannon diversity and evenness index (Additional file 1: Appendix A).

### Spatial variation: bacterial community composition in the guts of *C. palumbus* weevils at different sites

Plant species had no significant effect on *C. palumbus* weevil gut bacterial community composition (PERMANOVA: *F*_*1,52*_ = 1.05, *R*^*2*^ = 0.02, *P* = 0.329). In addition, there was no effect of the interaction between the sampling site and plant species (PERMANOVA: *F*_*3,52*_ = 1.10, *R*^*2*^ = 0.05, *P* = 0.299). Therefore, the data for the host plants *S. inermis* and *S. incanescens* were pooled for visualization and interpretation of the effects of geographical location on bacterial community composition.

**Fig. 3:**
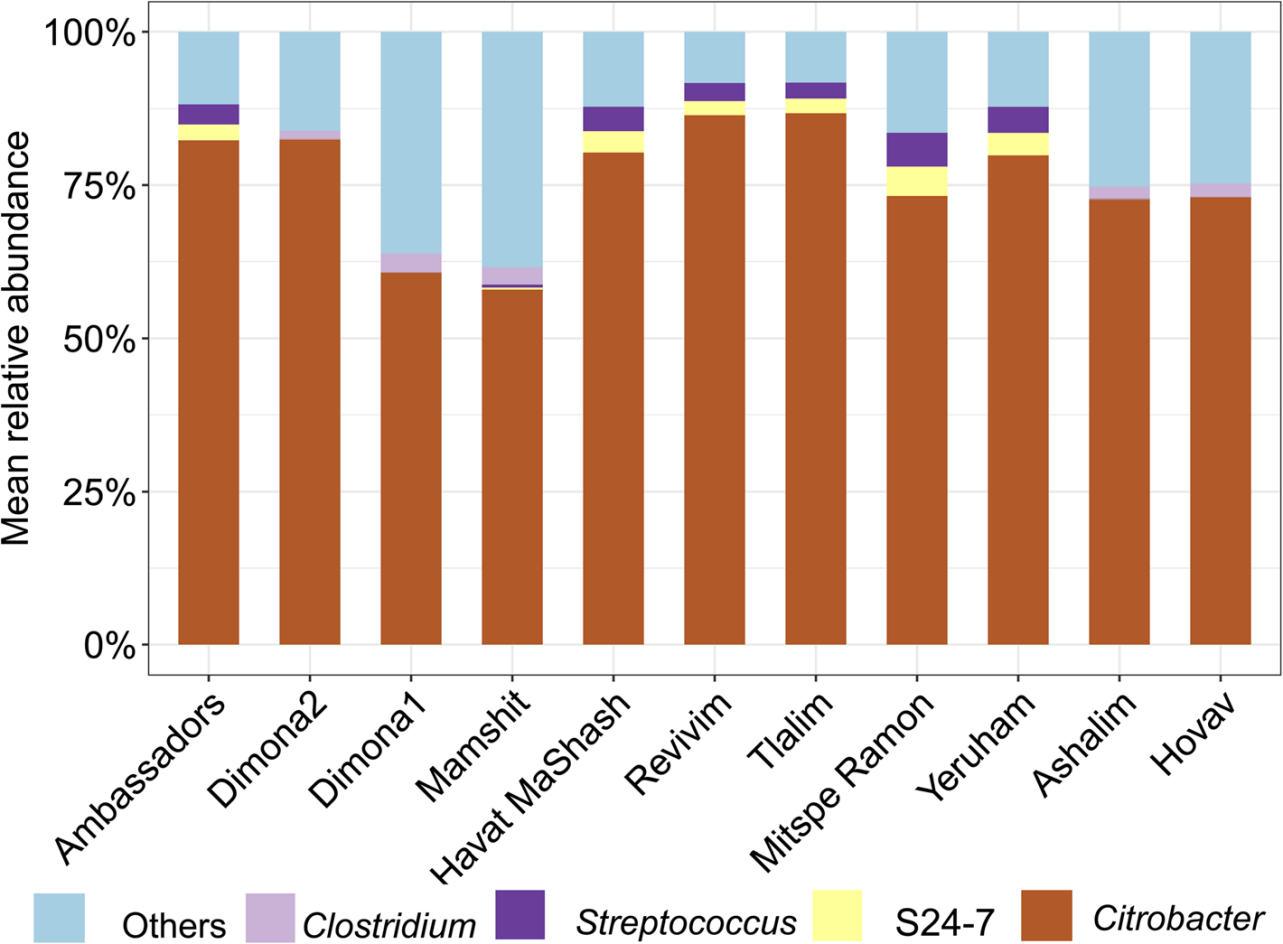
Mean relative abundance of bacterial genera in the guts of *Conorhynchus palumbus* larvae across 11 different sites (Ashalim: *N* = 6; Abu Haduba, Dimona2, Dimona1, Mamshit, Havat MaShash, Tlalim, Yeruham, and Neot Hovav: *N* = 5; Mitzpe Ramon: *N* = 4; Revivim: *N* = 3). S24–7 was provided because the phylotype was not classified to lower taxonomic level. Only those genera with > 1% mean relative abundance across all weevil samples are shown, whereas all remaining sequences are represented as others

PERMANOVA based on unweighted UniFrac distance (PERMANOVA: *F*_*10,52*_ = 6.22, *R*^*2*^ = 0.60, *P* = 0.740) showed that *C. palumbus* weevil gut bacterial communities were similar across locations (Fig. 3). Results based on binary Jaccard index, abundance Jaccard index and weighted UniFrac distance (Additional file 1: Table S1), as well as results based on relative abundance of genus (PERMANOVA: *F*_*10,52*_ = 2.24, *R*^*2*^ = 0.35, *P* = 0.154), showed a similar pattern. The communities were predominantly (at all sites) composed of *Citrobacter*, followed by *Streptococcus*, S24–7, and *Clostridium*. Again, the predominancy of *Citrobacter* in *C. palumbus* was mainly driven by the high relative abundance of a single OTU that was present in all individual *C. palumbus* weevils with a mean relative abundance of 71.52 ± 2.53%. The relative abundance of *Citrobacter* did not differ across locations (one-way ANOVA: *F*_*10,52*_ = 0.79, *P* = 0.636).

### Temporal variation: bacterial community composition in the guts of *C. palumbus* weevils at different developmental stages

**Fig. 4:**
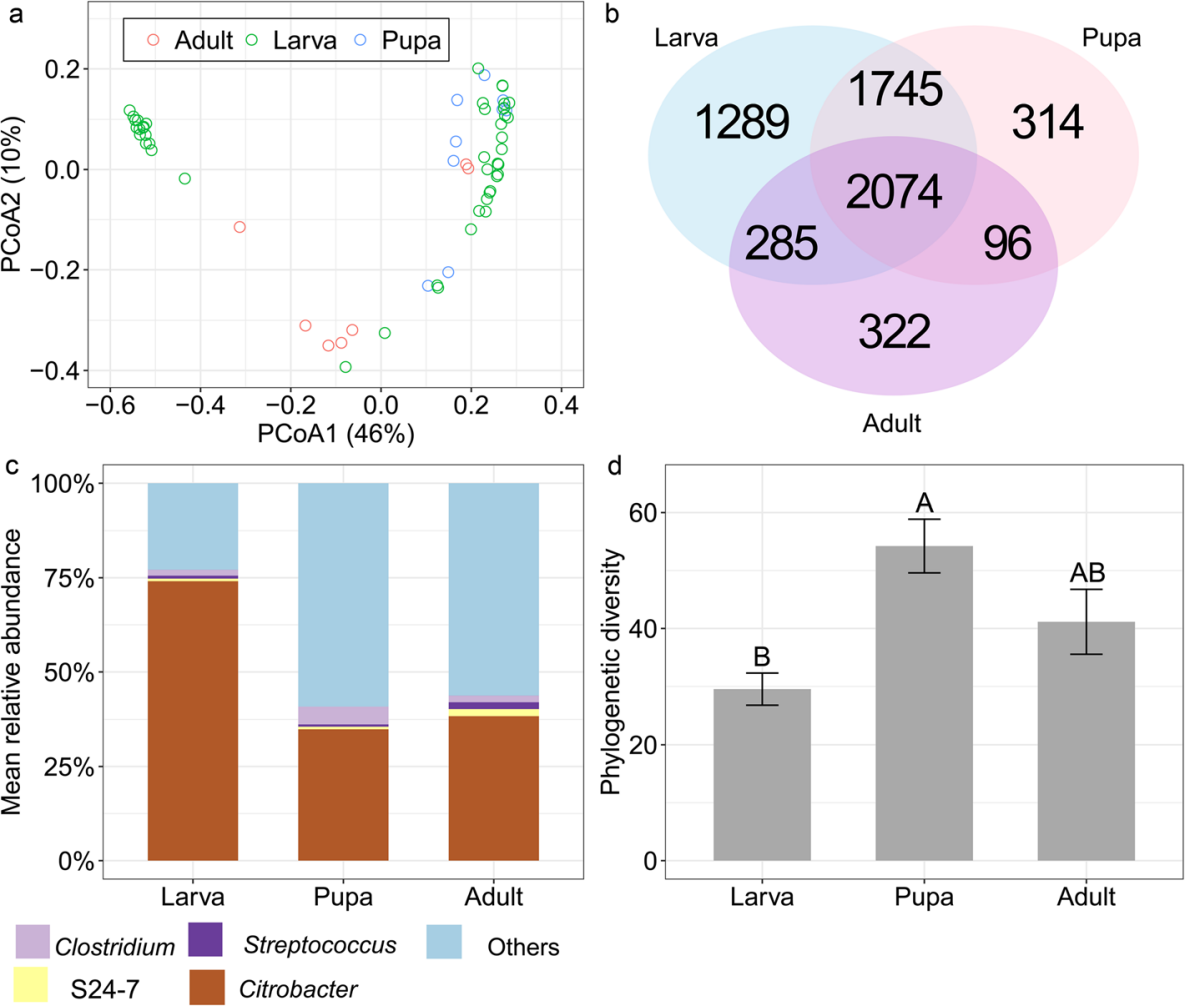
Bacterial community composition in the guts of *Conorhynchus palumbus* at different developmental stages (larva: *N* = 44, pupa: *N* = 9, adult: *N* = 7). (**a**) PCoA plot displaying unweighted UniFrac distance. The percent variation explained by each principle coordinate is shown. (**b**) Venn diagram representing number of OTUs that are unique to each of developmental stage and shared between them. (**c**) Mean relative abundance of bacterial genera. Only those genera with > 1% mean relative abundance across all samples are shown, whereas all remaining sequences are represented as others. S24–7 was provided because the phylotype was not classified to lower taxonomic level. (**d**) Phylogenetic diversity. Columns with different letters are different at *P* < 0.05 based on Tukey’s post hoc tests

PERMANOVA and PCoA based on unweighted UniFrac distance showed that the bacterial communities significantly differed among weevil life stages (PERMANOVA: *F*_*2,59*_ = 2.51, *R*^*2*^ = 0.08, *P* = 0.017; Fig. 4a,b; see Additional file 1: Appendix A for results of equivalent tests). Post hoc pairwise tests indicated no significant difference in bacterial communities between guts of larvae and those of pupae (*P* = 0.222) and adults (*P* = 1.00), while there was a significant difference in the gut bacterial communities of pupae and adults (*P* = 0.015).

The PCoA plot further indicated two separate clusters of larvae. To understand what accounts for these differences we compared larval mass and date of collection between larvae of the two clusters. We found that larvae that clustered alone were those collected earlier in the season (before mid-August), and were smaller in size (fresh biomass: 155.1 ± 31.2 mg, *N* = 15), while larvae that clustered more closely with pupae and adults were those collected later in the season (after mid-August), and were larger in size (fresh biomass: 277.9 ± 23.5 mg, *N* = 29; *t*-test comparing larval mass: *t*_*43*_ = 42, *P* = 0.004), suggesting they were closer to pupation.

The bacterial communities significantly differed among weevil life stages also at the genus level (PERMANOVA: *F*_*2,59*_ = 8.13, *R*^*2*^ = 0.22, *P* = 0.001; Fig. 4c). In this case, bacterial communities from the guts of larvae significantly differed from those of pupae (*P* = 0.018) and adults (*P* = 0.033), while there was no significant difference in the gut bacterial communities of pupae and adults (*P* = 0.414).

A similarity percentage analysis revealed four bacteria contributing to 50% of the dissimilarity between the adults and the larvae, including *Citrobacter* (contributing to 41% of the dissimilarity) being more abundant in the larvae, and *Acinetobacter*, *Massilia*, and *Clostridium* being more abundant in the adults (Table 2). A similarity percentage analysis between the guts of larvae and pupae showed that two genera contributed to 50% of the dissimilarity, including *Citrobacter* being more abundant in the larvae (contributing to 46% of the dissimilarity), and *Clostridium* being more abundant in the pupae (Table 2). Consistently, a significant decrease in the relative abundance of *Citrobacter* at pupal and adult stages (GLMM: *F*_*2,59*_ = 10.70, *P* < 0.001) was detected. This was mainly driven by the high abundance of a single OTU that was present in all individual *C. palumbus* weevils with a mean relative abundance of 71.34 ± 3.91%, 31.64 ± 10.7%, and 32.56 ± 9.86% in the larvae, pupae, and adults, respectively.

**Table 2:** Summary of bacteria that contributed to 50% of the dissimilarity between the larva and the adult, and between the larva and the pupa, revealed by similarity percentage analyses. + represents more abundant, − represents less abundant

Larva vs. Adult				Larva vs. Pupa			
Bacteria	%Contribution	Larva	Adult	Bacteria	%Contribution	Larva	Pupa
*Citrobacter*	41.01	+	–	*Citrobacter*	46.01	+	–
*Clostridium*	1.82	–	+	*Clostridium*	3.57	–	+
*Acinetobacter*	5.1	–	+				
*Massilia*	2.77	–	+				

The weevil at the larval stage had the lowest alpha diversity based on phylogenetic diversity, while there was no significant difference between weevil pupal and adult stages (GLMM: *F*_*2,59*_ = 8.20, *P* < 0.001; Fig. 4d). Similar results were obtained when comparing the Chao1 richness index, Shannon diversity and evenness index (Additional file 1: Appendix A).

### Bacterial community composition in the guts of the different weevil species

**Fig. 5:**
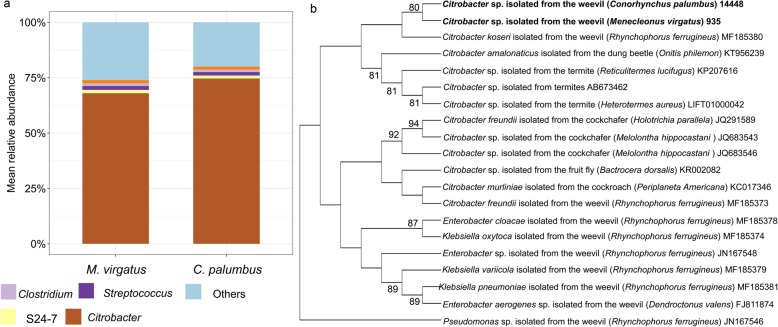
(**a**) Mean relative abundance of bacterial genera in the guts of weevil larvae *Conorhynchus palumbus* (*N* = 88) and *Menecleonus virgatus* (*N* = 8). (**b**) Neighbour-Joining phylogenetic tree constructed from MUSCLE alignment of the V4 region of the 16S rRNA gene of two dominant representative *Citrobacter* sequences obtained in this study, different *Citrobacter* sequences obtained from a range of insects, and representative *Enterobacter* and *Klebsiella* sequences (of the Enterobacteriaceae family) obtained from the other weevil species in MEGA v10.0. *Pseudomonas* sp. (of the Pseudomonadaceae family) was used as the out group. Genbank accession No. is shown for each sequence obtained from Genbank, while the OTU No. (the same as in the deposited datasets in Genbank) is shown for the sequence obtained in this study. Numbers on the branches are bootstrap values. Only bootstrap values greater than 70 are shown. Sequences in bold were obtained in this study

PERMANOVA based on unweighted UniFrac distance (PERMANOVA: *F*_*1,95*_ = 2.9, *R*^*2*^ = 0.02, *P* = 0.093; see Additional file 1: Table S1 for results of equivalent tests) and on relative abundance of genus (PERMANOVA: *F*_*1,95*_ = 0.49, *R*^*2*^ = 0.01, *P* = 0.601) indicated no significant difference in the gut bacterial community compositions of *C. palumbus* and *M. virgatus* (Fig. 5a). The gut bacteria of the two weevil species were dominated by *Citrobacter*, followed by *Streptococcus*, S24–7, and *Clostridium*. However, *C. palumbus* and *M. virgatus* differed in their dominant OTUs (both of which were also present but were less abundant in mud chambers and surrounding soils). Specifically, the mean relative abundance of the dominant OTU (classified as *Citrobacter*) present in *C. palumbus* was 71.73 ± 1.91%, while its relative abundance was 0.15 ± 0.87% in *M. virgatus*. Similarly, the mean relative abundance of the dominant OTU (also classified as *Citrobacter*) present in *M. virgatus* was 66.63 ± 3.84%, while its relative abundance was 0.67 ± 0.20% in *C. palumbus*.

The phylogenetic tree constructed by Neighbour-Joining analyses of the V4 region of the 16S rRNA gene showed that the representative dominant *Citrobacter* sequences isolated from *C. palumbus* and *M. virgatus* were phylogenetically close to each other and were the closest to *C. koseri* isolated from the palm weevil (*Rhynchophorus ferrugineus*) (Fig. 5b).

## Discussion

The objective of this study was to characterize the gut bacteria of weevils associated with *Salsola* plants, as a first step to address the hypothesis that these bacteria are involved in the adaptation of insects to extreme desert environments. Our results revealed high stability of bacterial community composition among weevils of the different species, geographical locations, and host plants.

Several gut bacteria have been found to be associated with weevils in this study, including the predominant *Citrobacter* (of the Enterobacteriaceae family), as well as the less abundant *Clostridium, Streptococcus*, and the Bacteroidales family S24–7. Our phylogenetic analysis indicated that *Citrobacter* in our weevils was most closely related to strains isolated from the palm weevil. *Citrobacter* have been previously reported to dominate in several species of weevil beetles, e.g.*,* the palm weevil, *R. ferrugineus*, and the bark beetle, *Dendroctonus armandi* [32, 35, 40–42], whereas it was less abundant in others, e.g.*,* the bark beetles, *D. valens* and *D. mexicanus* [43]. Other than in weevils, *Citrobacter* has been commonly reported to form associations with a variety of insects, such as termites, flies and moths (see literature survey, Additional file 1: Appendix C). The occurrence of *Citrobacter* in the gut flora of insects of different orders may suggest its general role in supplementing their diets. Indeed, previous studies have reported that different members of *Citrobacter* are associated with nitrogen fixation, uric acid recycling, and cellulose degradation in the guts of different insects (Additional file 1: Appendix C) [12, 44–48]. In addition, different members of *Citrobacter* from soils are commonly associated with other processes of nitrogen metabolism, e.g.*,* denitrification and nitrification [49, 50]. Nitrogen fixation has been previously demonstrated to occur in *C. palumbus* larvae [23]. Although *Klebsiella* spp. was suggested as the main nitrogen-fixing bacteria, *Citrobacter* could also play a role in this function [24]. Uric acid recycling by *Citrobacter* may also potentially be important for the nitrogen budgets of weevils in our study system. Cellulose degradation, however, is less likely to occur as the weevil larvae do not feed on plant tissues [25].

Although in lower relative abundance than *Citrobacter*, other bacterial groups may have potential impacts on the weevils. For example, the genus *Clostridium* that is widely distributed in soils, and in the guts of humans and animals, and have also been reported at a low relative abundance in few species of weevil beetles [41, 42], has been shown to be able to ferment complex molecules including cellulose, hemicellulose, and pectin [51]. In addition, the Bacteroidales family S24–7 has been commonly found within murine gut, where it contributes to carbohydrate degradation [52], as well as within insect gut where its function has not yet been characterized [53]. Different members of *Streptococcus* occur mostly in human mouth and respiratory tracts, and less commonly in soils [54], but have been occasionally found in insects as potential pathogens [55], as well as in other weevil species where its function has not yet been characterized [42, 56–58]. As such, these less common bacterial genera could also have an impact on the diet and the health of the weevils.

We found no evidence of the *Nardonella*—the most ancient and widespread bacterial endosymbiont in weevils of the superfamily Curculionoidea)—nor of its alternate clades (e.g.*, Sodalis* and *Curculioniphilus*) [59], although the location of these bacteriome-localized bacteria is often closely associated with the larval gut [59, 60]. The absence of *Nardonella* from our samples concurs with previous evidence that *Nardonella* is absent from weevils of the sub-family Lixinae [61], as well as from weevils of other groups (e.g.*,* [30]). Notably, similar to our results, in the weevil *Irenimus aequalis*, *Nardonella* was absent, and the dominant bacterium was a member of the Enterobacteriaceae family [30].

Bacterial composition in the weevil guts was associated with the weevil developmental stage. Similarly, previous studies show changes in the bacterial community composition throughout the insect host’s life cycle in response to diet shifts or to changes in internal morphology and physio-chemical conditions inside the insect [62, 63]. In fact, changes (either an increase or decrease) in bacterial diversity and shifts in the dominant gut bacteria across insect life stages seem to be the norm [3, 62–65]. For example, shifts in the dominant bacteria from Proteobacteria in the larvae (leaf chewer) to Firmicutes in the pupae and the adults (nectar feeder), accompanied with changes in gene expression, were demonstrated in the cotton leafworm [63]. In other cases, a change in bacterial diversity occurs while the dominant bacteria remain consistent, as shown in a forest cockchafer, in which the gut bacteria of the diapausing adults represented a subset of those of the larvae [66]. In our study system, the higher community diversity in the pupae and adults, coupled with the decrease in the relative abundance of the predominant group *Citrobacter*, may be due to the fact that the gut is renewed during metamorphosis [67]. More specifically, these changes could be associated with the absence of feeding activity at these stages, as the weevil’s gut is emptied in the pupal stage (pers. obs. by F. Meng), and possibly already at late larval stages, and the adults sampled in this study were collected while diapausing.

The stable gut bacterial community of the weevils across a wide geographic range and host plants implies the functional importance of the dominant taxa. This notion is supported by a number of studies that have demonstrated high stability of functionally relevant microbial communities in different insects, including other species of weevil beetles [38, 41, 68]. For example, in the wood-feeding pine and palm weevils, gut bacteria exhibited a highly stable microbial community with high prevalence of the Enterobacteriaceae across different locations, and the Enterobacteriaceae members were demonstrated to detoxify toxins and degrade cellulose in the weevils’ diets [32, 36, 37]. In contrast, other studies demonstrated large spatial variation in bacterial communities, possibly resulting from geographic isolation [69] and variation in external environmental conditions. For example, in the black chafer beetle, the variation in bacterial communities across geographic locations was related to climatic factors and soil properties [65].

A high proportion of bacteria OTUs (including all weevil core OTUs) observed in the weevil guts were also observed in the environment, suggesting that the weevils may potentially acquire most of their gut bacteria from the soil. However, the pronounced differences in bacterial composition between the weevils and the soil (as apparent in the PCoA plots), combined with the high stability of the gut bacterial community (especially of the core bacteria) of the weevils, suggests that internal factors in the weevil guts are more important than external environmental factors, in shaping it. In particular, the reduction in the diversity of bacterial species in weevil guts, relative to their surroundings, could be attributed to the selection of specific bacterial populations, mediated via weevil gut morphology and/or physiology, as demonstrated in other insects, including termites [70] and the bean bug [71]. Consistently, the high relative abundance and persistence of *Citrobacter* and, particularly, of a specific OTU may suggest that *Citrobacter* could be selected by the weevil internal environment to support weevil nutrition. The fact that *Citrobacter* was common in *C. palumbus* and *M. virgatus*, but was represented by a different OTU in each species may suggest that the weevils acquire it vertically, as suggested for fruit flies [72]. However, this may also be explained by horizontal transmission [73], combined with differences in the internal morphology or physiological environment of the two weevil species, potentially selecting for different *Citrobacter* strains [70], especially given that both OTUs were also found in the surrounding environments in this study and that *Citrobacter* members are widely distributed in soils [49, 74]. Further investigations are needed to clarify the function and mode of the transmission of *Citrobacter* associated with these weevils.

In view of the extremely low nitrogen content of *Salsola* plants, we suggest that gut bacteria could contribute to the fitness of weevils in confronting their challenging nutritional environment. Similarly, the desert locust, which is widely distributed in the desert regions of northern Africa, the Middle East, and southwest Asia, contained a relatively simple but abundant gut bacteria including different members of Enterobacteriaceae [75–78]. The persistence of these bacteria in the guts of the desert locust was suggested as an adaptation to overcome the limited nitrogen in this environment. Additional studies on insect gut bacteria in desert ecosystems, combining metagenomic, metatranscriptomic, biochemical analyses and experimentation, are crucial to determine the generality of such interactions and their adaptive role under extreme climatic conditions.

## Conclusions

The study shows highly stable gut bacterial community and predominancy of the genus *Citrobacter* in weevils across a wide geographical range in the Negev Desert, suggesting a major role for *Citrobacter* in weevil nutrition. This is one of very few studies that explicitly examine insect gut bacteria in the desert, highlighting the potential involvement of such bacteria in the adaptation of insects to extreme environmental conditions.

## Methods

### Study area

The study was conducted in the Negev Plateau in Israel’s Negev Desert (30°38′49.3″~ 31°18′50.5″N, 34°40′22.2″~ 35°03′00.2″E). The average maximum daily temperature is ~ 33 °C in July–August, and the annual rainfall ranges from 34 to 187 mm with a mean of ~ 100 mm, with all rainfall occurring during the winter months from November to April; hence, the summers in this region are characterized as hot and dry (the Israel Meteorological Service; http://www.ims.gov.il/IMSEng/CLIMATE).

### Sample collection

To characterize the spatial variation of gut bacteria, we collected weevil, soil, and mud chamber samples in 11 sites (Additional file 1: Appendix D) during August 2017, when the beetles were at their larval stage. To characterize changes in gut bacterial community composition throughout the weevil life cycle, samples were collected monthly from two sites: Ashalim (dominated by *S. incanescens*) and Neot Hovav (dominated by *S. inermis*), starting from July 2017 (early larval developmental stage) till November 2017 (weevil diapausing adult stage when the adult is still inside the mud chamber underground), for a total of five sampling events for each site.

In each site, we excavated *Salsola* plant roots to collect 3–10 weevils (according to availability) together with their mud chamber. In addition, we took a soil sample from a depth of ~ 10 cm (typical depth of the weevil mud chamber) from the rhizosphere of each individual *Salsola* plant. We cleaned the digging tools, tweezers, and gloves with 75% ethanol between each sample collection. All the samples were transported to the laboratory in sterile vials in the icebox within 6 h and immediately stored at − 80 °C for subsequent DNA extraction.

A total of 72 weevils (including 49 larvae, 10 pupae, and 13 adults) were sampled to characterize temporal variation in bacterial composition throughout the weevil life cycle, and a total of 56 weevil larvae (including 11 larvae which were also used for temporal variation) were sampled to characterize spatial variation in bacterial composition. According to a previous survey, three weevil species develop affixed to the roots of *Salsola* plants in the Negev Desert [24], and hence, we used genetic markers to identify the weevil larvae and pupae to species (see below), while all the sampled weevil adults were morphologically identified as *C. palumbus*. Since many mud chambers broke during the excavation process, we only used intact ones (given that we were specifically interested in the bacteria of the mud chambers’ inner layers) to avoid potential contamination with associated surrounding soils. This resulted in a total of 25 intact mud chamber samples and 25 surrounding soil samples used for subsequent analyses.

### Weevil dissection and DNA extraction

Prior to the dissection, the samples were washed in three different solutions: 1% dish soap, 100% ethanol, and phosphate buffer pH 7.0 (Caisson Labs, Smithfield, UT, USA), and this procedure was repeated three times [47]. The weevils were dissected with the aid of insect pins to excise the whole gut. The gut was transferred to a 2-mL sterilized microcentrifuge tube with 3-mm and 40-μm radius glass beads, and 100 μl of EDTA pH 8.0 (50 mM, bioWORLD, Dublin, OH, USA) was added. Tweezers and pins were cleaned with 70% ethanol and fire between each dissection. All the solutions were sterilized, and all the procedures were performed in a sterile environment.

For larval lysis, samples were vortexed at maximum speed for 30 s, followed by 30 s of freezing in liquid N_2_; this procedure was repeated three times. The homogenized samples were incubated for 1 h at 37 °C with 120 μl of lysozyme (10 mg/ml, Amresco, Solon, OH, USA). Samples were then centrifuged at 13,000 g in 4 °C for 10 min, and the supernatant was removed to allow DNA purification. DNA was then extracted using a Wizard Genomic DNA Purification Kit (Promega, Madison, WI, USA) according to the manufacturer’s protocols. This Purification Kit was used after comparing the DNA extraction efficiency of two widely used commercial kits (i.e.*,* Wizard Genomic DNA Purification Kit and Qiagen DNeasy Blood & Tissue Kit).

### Soil and mud chamber DNA extraction

Mud chamber samples consisted of 1 g of soil taken from the internal portion of the mud chamber. The soil sample was homogenized in a mortar and pestle with liquid N_2_, and DNA was extracted from 1 g of subsamples of each soil sample. DNA was extracted using the PowerSoil DNA Isolation Kit (MO BIO, Carlsbad, CA, USA) according to the manufacturer’s protocols. This DNA Isolation Kit is widely used and highly recommended for DNA extraction from environmental samples (including soil) [79].

To ensure sample quality, PCR amplification of the entire 16S rRNA gene (with a positive control and water as a negative control) was conducted for each sample after DNA extraction, following a general PCR protocol [80]. No PCR products were detected for any of the negative controls while there were clear bands for positive controls on a 2% agarose gel.

### Weevil species identification

We amplified the gene cytochrome c oxidase subunit I to identify weevil larvae and pupae to species, as previously described [24]. Briefly, PCR was performed in a 20-μl reaction volume, containing 10 μl Taq Bio-ReadyMix (Bio-Lab, Jerusalem, Israel), 1 μl template, 6.6 μl DNase-free water, 0.7 μl of each primer for *C. palumbus* (forward:5′- TTAGTCCCTCTCATACTAGGAGCC − 3′, reverse: 5′- GAAGAGAAAGAAGGAGTAAAATAGCGG − 3′), and 0.5 μl of each primer for *M. virgatus* (forward:5′- ACTTCCGCCATCTTTAACCTTGT − 3′, reverse: 5′- GGTAGTTCGGTCAGGTGT − 3′). The cycling parameters were: initial denaturation at 95 °C for 5 min, followed by 40 cycles of denaturation at 95 °C for 30 s, annealing at 55 °C for 30 s and extension at 72 °C for 30 s, and a final extension at 72 °C for 5 min. We identified 108 *C. palumbus* (a PCR product size of 354 bp) including 74 collected from *S. inermis* and 34 collected from *S. incanescens*, and eight *M. virgatus* (a PCR product size of 235 bp) including two collected from *S. inermis* and six collected from *S. incanescens* from all samples.

### Identification and taxonomic classification of 16S rDNA fragments

The Research Laboratory Hylab (Rehovot, Israel) conducted amplicon sequencing of the DNA samples. Briefly, 20 ng of metagenomic DNA was amplified in a 25 μl PCR reaction by PrimeStar Max DNA Polymerase (TAKARA Bio Inc., Otsu, Japan) for 20 cycles. The PCR products were purified using Ampure XP beads. Then the 2 μl of the first PCR was amplified to add the adaptor and index sequences in a 10 μl reaction for 10 cycles with the Fluidigm Access Array primers for Illumina. The PCR product was then purified and was sequenced on the Illumina MiSeq platform. The V4 region of the 16S rRNA gene was amplified using the universal bacterial forward primer 515f (5′-GTGYCAGCMGCCGCGGTAA-3′) and reverse primer 806r (5′-GGACTACNVGGGTWTCTAAT-3′), and 250 bp paired-end reads were generated.

Sequence processing was done by using the bioinformatics platform QIIME v1.9.1 [81]. We removed sequencing reads if they contained ambiguities or homopolymers (> six nucleotides in length), or if the average Phred quality score was less than 25 using the default setting in QIIME. Primer sequences were trimmed, and chimeric sequences were eliminated using USEARCH (version 6.1) and the “gold” reference database [82]. Sequences that passed these quality filters were classified into OTUs using the de novo clustering method at 97% similarity with USEARCH and nontarget reads (i.e., chloroplasts and unclassified) were removed. Representative OTUs were then aligned against the SILVA bacterial database (https://mothur.org/wiki/Silva_reference_files) with a threshold confidence level of 50%, and taxonomic classification was carried out with Mothur (version 1.36.1) using the Wang approach [83].

Our samples resulted in 4,384,135 quality sequences averaging 26,410 sequences per sample (ranging from 66 to 72,207). Of the initial 167 samples (including 117 weevil, 25 mud chamber, and 25 surrounding soil samples), we failed to detect bacterial DNA in one weevil pupa sample, and an additional six adult weevil samples yielded < 3700 sequences (ranging from 66 to 1275 sequences). This led to a total of 160 samples that were used for downstream analyses, corresponding to 10,220 OTUs: ranging from 158 to 2904 OTUs, with an average of 987 per sample for weevils, 1986 per sample for mud chambers, and 2557 per sample for the surrounding soil (see Additional file 1: Appendix E for full details on sample sizes used for analyses and sequencing results).

### Data analysis

We tested whether the different sample types (i.e.*,* weevil, mud chamber and surrounding soil), weevil species (i.e.*, C. palumbus* and *M. virgatus*), host plant species (i.e.*, S. inermis* and *S. incanescens*), weevil developmental stages (i.e.*,* larva, pupa and adult), and sampling sites, differed in their presence/absence of OTUs using binary Jaccard index (taxonomic) and unweighted UniFrac distance (phylogenetic), as well as relative abundances of OTUs using abundance Jaccard index (taxonomic) and weighted UniFrac distance (phylogenetic) with QIIME v1.9.1. The abundance Jaccard index and weighted UniFrac distance were generated from a CSS normalized OTU table with QIIME v1.9.1. The differences in microbial community composition at higher level were also assessed based on the relative abundances of genera. The statistical significance in these comparisons were assessed by using PERMANOVA as implemented in the “adonis” function in the R package vegan [84]. Pairwise comparisons for significant PERMANOVA results were conducted and corrected for multiple testing using a Bonferroni correction as implemented in the “pairwise.adonis” function in the R package pairwiseAdonis [85]. The observed dissimilarity for significant pairwise comparison results was assessed using similarity percentage analyses as implemented in the “simper” function in the R package vegan. PCoA were carried out in QIIME to visualize the significantly different dissimilarity matrices. The individual plant ID was regarded as a random effect (i.e.*,* specified “strata = individual plant ID” in the adonis test) when differences in microbial community composition among different sample types (weevil, mud chamber, and soil) were assessed, while sampling site was regarded as a random factor (i.e.*,* specified “strata = sampling site” in the adonis test) when differences across weevil life cycle were assessed. Results of binary Jaccard index, abundance Jaccard index, unweighted and weighted UniFrac distance were similar, and hence we present only the result of unweighted distance and the results of others are presented in the supplementary material.

In addition, we tested whether sampling sites and weevil developmental stages differed in relative abundances of the predominant genus *Citrobacter*. Plant species had no significant effect on the relative abundance of *Citrobacter*, and hence the data for the host plants *S. inermis* and *S. incanescens* were pooled for subsequent analyses. For spatial variation, one-way ANOVA was then conducted to assess the effect sampling sites on the relative abundance of *Citrobacter*, while, for temporal variation, GLMMs with sampling site as a random factor, followed by Tukey’s post hoc tests, were performed to examine the effects of weevil developmental stage on the relative abundance of *Citrobacter*.

Given that we only identified eight *M. virgatus* weevils, these were used for an analysis on the differences between weevil species, while all other analyses (including the differences between different sampling types, sampling sites, and weevil developmental stages) were based only on the common weevil species *C. palumbus*. In addition, only *C. palumbus* larvae were used for the comparison between weevil species, provided that only *M. virgatus* larvae were found.

We calculated alpha diversity by performing phylogenetic diversity (estimates using the PD Whole Tree), Chao1 richness (expressed as the number of observed unique OTUs) and Shannon diversity estimates in QIIME. Evenness index was also calculated as: Evenness index = Shannon diversity/ln (the number of observed OTUs derived for each sample). Prior to alpha diversity calculation, we rarefied OTU tables in QIIME such that all samples were randomly rarefied to 3700 sequences (the lowest number among all samples) per sample such that they had equal sampling effort, thus removing heterogeneity among samples. We used GLMMs with individual plant ID and sampling site as random factors, followed by Tukey’s post hoc tests, to examine the effects of sample type and weevil developmental stage, respectively, on the alpha diversity. The results of Chao1 richness index, Shannon diversity, evenness index and phylogenetic diversity were similar, and hence we present only the result of phylogenetic diversity and the results of others are presented in the supplementary material.

## Supplementary information

**Additional file 1.** Appendix A Bacterial communities of different sample types and in the guts of *C. palumbus* weevils at different developmental stages. Appendix B. Assessment of the representativeness of soil bacterial communities in Negev Desert. Appendix C. Suggested functions of different species of *Citrobacter* from a wide range of insects. Appendix D: Location of sampling sites. Appendix E. Sampling and sequencing details of all analyzed samples

## Data Availability

The sequence data that support the findings of this study were deposited into Targeted Locus Study project at GenBank under the accession KDDD00000000 (https://www.ncbi.nlm.nih.gov/nuccore/KDDD00000000). The version described in this paper is the first version, KDDD01000000.

## Abbreviations

GLMM: Generalized linear mixed model
OTU: Operational taxonomic unit
PCoA: Principal coordinates analysis
PERMANOVA: Permutational multivariate ANOVA
