# Supplementary material for: Gut bacteria of weevils developing on plant roots under extreme desert conditions
Source: BMC Microbiol. 2019 Dec 30;19:311. doi: 10.1186/s12866-019-1690-5 (PMC6937996; doi:10.1186/s12866-019-1690-5)
Supplement: Supplementary file 1 — Additional file 1. Appendix A Bacterial communities of different sample types and in the guts of C. palumbus weevils at different developmental stages. Appendix B. Assessment of the representativeness of soil bacterial communities in Negev Desert. Appendix C. Suggested functions of different species of Citrobacter from a wide range of insects. Appendix D: Location of sampling sites. Appendix E. Sampling and sequencing details of all analyzed samples [file 12866_2019_1690_MOESM1_ESM.docx]

**Supplementary materials**

**Appendix A:** Bacterial communities of different sample types and in the guts of *C. palumbus* weevils at different developmental stages

*Bacterial communities of different sample types*

PERMANOVA and PCoA based on binary Jaccard index, abundance Jaccard index and weighted UniFrac distance showed that weevils, mud chambers, and soils significantly differed in their bacterial communities (Table S1; Fig. S1 a-c). Post hoc pairwise tests showed that bacterial communities from the weevil guts were significantly different from those of the mud chambers and of the surrounding soils; and that those of mud chambers were also significantly different from those of the surrounding soils (Table S1).

Weevil gut bacteria had the lowest alpha diversity based on Chao1 richness, Shannon diversity and evenness index (Fig. S1 d-f).

**Table S1:** Summary of PERMANOVA analyses and post hoc pairwise comparisons examining differences in the bacterial communities of different sample types (*N* = 25 for each of sample type) and weevil developmental stages (*N* = 44 for larva, *N* = 9 for pupa, and *N* = 7 for adult). Significant difference is shown in italics and bold.

| Factor | Binary Jaccard | |  | Abundance Jaccard | |  | Weighted UniFrac | |
| --- | --- | --- | --- | --- | --- | --- | --- | --- |
|  | *F* | *P* |  | *F* | *P* |  | *F* | *P* |
| Sample type | 18.6 | ***0.001*** |  | 40.5 | ***0.001*** |  | 123.8 | ***0.001*** |
| larva-soil | 515.1 | ***0.003*** |  | 786.2 | ***0.003*** |  | 971.8 | ***0.003*** |
| larva-chamber | 326.2 | ***0.003*** |  | 465.3 | ***0.003*** |  | 639.3 | ***0.003*** |
| soil-chamber | 4.6 | ***0.003*** |  | 5.8 | ***0.006*** |  | 3.7 | ***0.003*** |
| Stage | 2.1 | ***0.011*** |  | 2.6 | ***0.017*** |  | 3.2 | ***0.023*** |
| larva-pupa | 3 | 0.225 |  | 3.3 | 0.252 |  | 3.4 | 0.249 |
| larva-adult | 0.4 | 1 |  | 0.3 | 1 |  | 0.01 | 1 |
| pupa-adult | 9.5 | ***0.009*** |  | 11.6 | ***0.003*** |  | 10.1 | ***0.003*** |
| Spatial | 4.2 | 0.75 |  | 7.7 | 0.755 |  | 23.2 | 0.752 |
| Species | 2.7 | 0.111 |  | 2.7 | 0.106 |  | 2.5 | 0.117 |


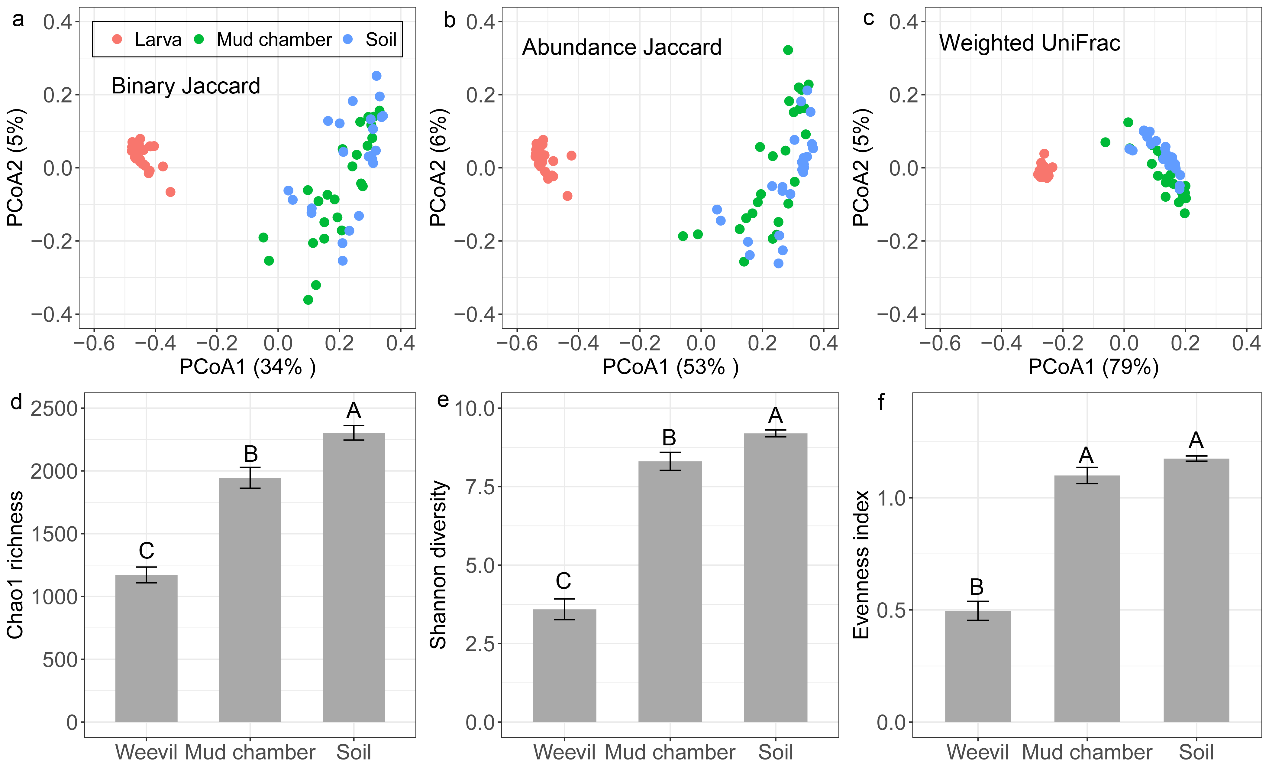


**Fig. S1:** Bacterial community compositions in the guts of *C. palumbus*, mud chamber and surrounding soil. PCoA plots displaying (a) binary Jaccard index, (b) abundance Jaccard index and (c) weighted UniFrac distance. The percent variation explained by each principle coordinate is shown. Alpha diversity based on (d) Chao1 richness index, (e) Shannon diversity, and (f) Evenness index. Columns with different letters are different at *P* < 0.05 based on Tukey’s post hoc tests.

*Bacterial communities in the guts of C. palumbus weevils at different developmental stages*

PERMANOVA and PCoA based on binary Jaccard index, abundance Jaccard index and weighted UniFrac distance showed that the bacterial communities significantly differed among weevil life stages (Table S1; Fig. S2 a-c). Post hoc pairwise tests showed that bacterial communities from the guts of larvae were similar to those of pupae and adults, while there was a significant difference in the gut bacterial communities of pupae and adults (Table S1). However, PCoA plot indicated no clear segregation of pupa samples and adult samples. There were two separate clusters of larvae that the smaller larvae clustered alone and the larger larvae clustered with the pupae and adults.

The weevil at the larval stage had the lowest alpha diversity based on Chao1 richness index, Shannon diversity and evenness index, there was no significant difference between weevil pupal and adult stages (Fig. S2 d-f).


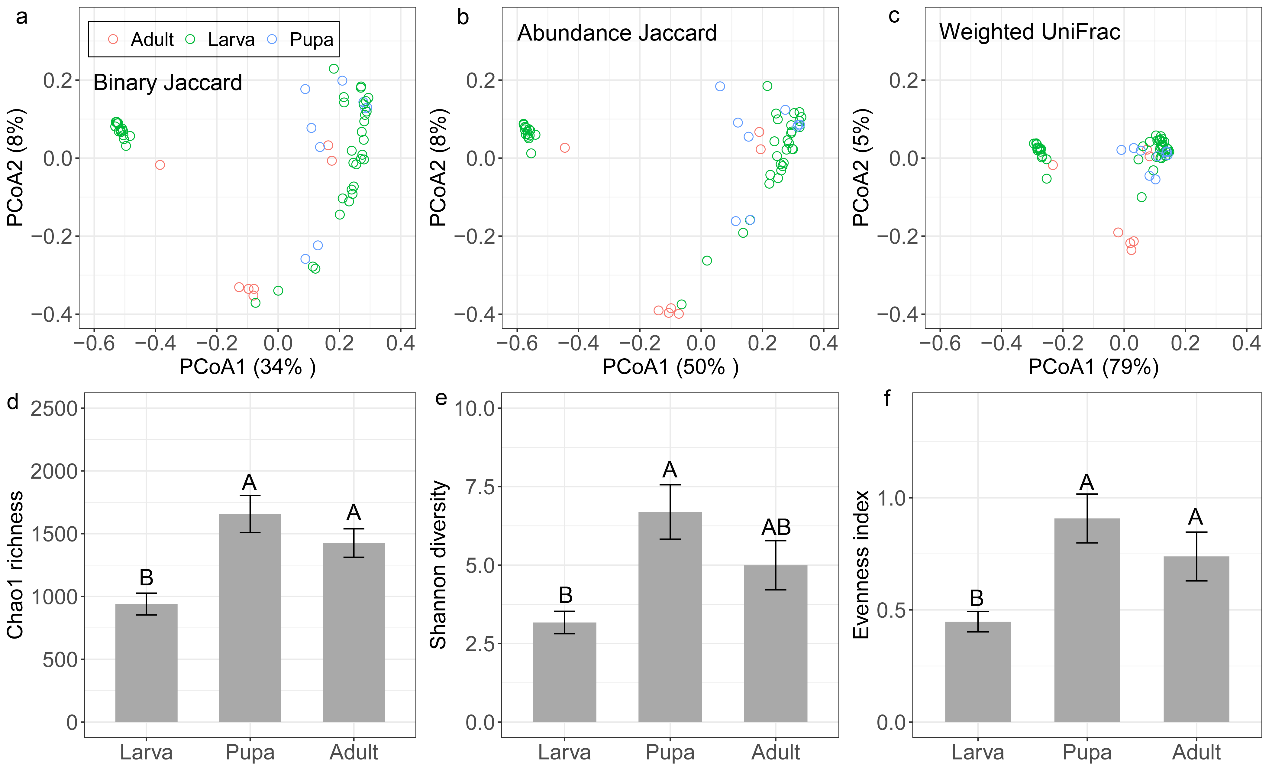


**Fig. S2:** Bacterial community composition in the guts of *C. palumbus* at different developmental stages. PCoA plots displaying (a) binary Jaccard index, (b) abundance Jaccard index and (c) weighted UniFrac distance. The percent variation explained by each principle coordinate is shown. Alpha diversity using (d) Chao1 richness index, (e) Shannon diversity, and (f) Evenness index. Columns with different letters are different at *P* < 0.05 based on Tukey’s post hoc tests.

**Appendix B: Assessment of the representativeness of soil bacterial communities in Negev Desert**

The total number of OTUs observed in the soil in our study (2,557 ± 113(SE)) was slightly higher than in the previous study (2,048 ± 118) (Baubin et al. 2019) in the same region of Negev Desert which used different DNA extraction kits and amplified different region of the 16S rRNA gene. Nevertheless, the soil bacterial communities were similar in these two studies (see Fig. S3 for this study and see Baubin et al. 2019 for the previosu study), both of which were dominated by the Actinobacteria (accounting for 36% and 27% of the relative abundance in soil, respectively) and Proteobacteria phyla (accounting for 24% and 30% of the relative abundance in soil, respectively), followed by Bacteroidetes, Chloroflexi and Planctomycetes phyla.


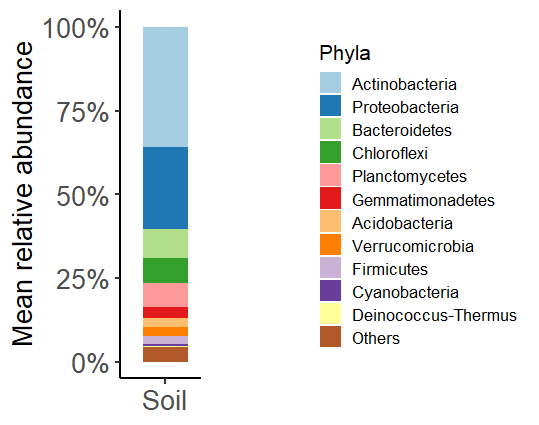


**Fig. S3:** Bacterial community compositions in soil (*N* = 25) in the Negev desert in this study

Reference

Baubin C, Farrell AM, Šťovíček A, Ghazaryan L, Giladi I, Gillor O. Seasonal and spatial variability in total and active bacterial communities from desert soil. Pedobiologia (Jena). 2019;74:7–14.

**Appendix C:** Suggested functions of different species of *Citrobacter* from a wide range of insects

**Table S2:** Suggested functions of different species of *Citrobacter* from a wide range of insects

| **Insect order** | **Insect species/family** | ***Citrobacter* species** | **Function** | **References** |
| --- | --- | --- | --- | --- |
| Blattodea | *Blatta orientalis* | *C. freundii* | Not determined | (1) |
| Blattodea | *Diploptera punctata* | *C. freundii* | Potential pathogen | (2) |
| Blattodea | *Periplaneta Americana*  *Blatta orientalis* | *C. freundii* | Potential pathogen | (3,4) |
| Coleoptera | *Cephaloleia* spp. | *Citrobacter* spp. | Not determined | (5) |
| Coleoptera | Passalidae  Staphylinidae  Scarabaeidae  Cerambycidae  Tenebrionidae  Elateridae | *Citrobacter* spp. | Not determined | (6) |
| Coleoptera | *Dendroctonus* spp. | *Citrobacter* spp. | Not determined | (7) |
| Coleoptera | *Aspidomorpha milliaris* | *Citrobacter* spp. | Detoxification of phytotoxins | (8) |
| Coleoptera | *Dendroctonus armandi* | *Citrobacter* spp. | Not determined | (9,10) |
| Coleoptera | *Dendroctonus micans* | *C. freundii* | Potential pathogen | (11) |
| Coleoptera | *Dendroctonus valens* | *Citrobacter* spp. | Not determined | (12) |
| Coleoptera | *Holotrichia parallela* | *C. freundii* | Cellulose degradation | (13) |
| Coleoptera | *Lepidiota mansueta* | *Citrobacter* sp. | Cellulose degradation | (14) |
| Coleoptera | *Leptinotarsa decemlineata* | *Citrobacter* spp. | Not determined | (15) |
| Coleoptera | *Oryctes agamemnon* | *C. freundii*  *C. youngae*  *C. murliniae* | Not determined | (16) |
| Coleoptera | *Oryctes monoceros* | *C. amalonacticus* | Not determined | (17) |
| Coleoptera | *Oryctes rhinoceros* | *Citrobacter* spp. | Cellulose and hemicellulose degradation | (18) |
| Coleoptera | *Rhynchophorus ferrugineus* | *C. freundii*  *C. koseri* | Cellulose degradation | (19,20) |
| Coleoptera | *Trogoderma granarium* | *Citrobacter* spp. | Not determined | (21) |
| Coleoptera | *Ips typographus* | *C. braakii*  *C. freundii* | Not determined | (15) |
| Coleoptera | *Melolontha hippocastani* | *Citrobacter* sp. | Xylan degradation | (22) |
| Coleoptera | *Onitis philemon* | *C. amalonaticus*  *C. freundii* | Cellulose and lignin degradation | (23) |
| Diptera | *Anastrepha ludens* | *Citrobacter* spp. | Not determined | (24) |
| Diptera | *Bactrocera dorsalis* | *C. freundii* BD | Trichlorphon degradation | (25) |
| Diptera | *Ceratitis capitata* | *C. freundii* | Nitrogen fixation | (26,27) |
| Diptera | *Lutzomyia longipalpis* | *C. freundii* | Not determined | (28) |
| Diptera | *Stomoxys calcitrans* | *C. freundii* | Oviposition stimulation | (29) |
| Homoptera | *Bemisia argentifolii*  *B. tabaci* | *Citrobacter* sp. | Honey dew production | (30) |
| Isoptera | *Reticulitermes speratus* | *C. freundii* | Not determined | (31) |
| Isoptera | Termites | *Citrobacter* spp. | Cellulose degradation | (32) |
| Isoptera | Termites | *Citrobacter* sp. | Uric acid degradation | (33) |
| Isoptera | *Coptotermes formosanus* | *Citrobacter* sp. SVUB3  *C. farmeri* | Not determined | (34–37) |
| Isoptera | *Coptotermes lacteus*  *Mastotermes darwiniensis*  *Nasutitermes exitiosus* | *C. freundii* | Nitrogen fixation | (38) |
| Isoptera | *Heterotermes aureus* | *Citrobacter* sp. CtB7.12 | Cellulose degradation | (39) |
| Isoptera | *Reticulitermes flavipes* | *Citrobacter* sp. | Uric acid degradation | (40–42) |
| Isoptera | *Reticulitermes lucifugus* | *C. farmeri* | Cellulose degradation | (43) |
| Isoptera | *Reticulitermes santonensis* | *Citrobacter* sp. | Not determined | (44) |
| Lepidoptera | *Automeris zugana* | *Citrobacter* spp. | Not determined | (45) |
| Lepidoptera | *Bombyx mori* | *C. freundii* | Cellulose and xylan degradation | (46) |
| Lepidoptera | *Helicoverpa armigera* | *Citrobacter* spp. | Not determined | (47) |
| Lepidoptera | *Hyphantria cunea* | *C. freundii* | Not determined | (48) |
| Lepidoptera | *Manduca sexta* | *C. sedlakii* | Not determined | (49) |
| Lepidoptera | *Pectinophora gossypiella* | *C. koseri* | Not determined | (24) |
| Lepidoptera | *Sesamia inferens* | *Citrobacter* spp. | Not determined | (50) |
| Lepidoptera | *Spodoptera littoralis* | *Citrobacter* spp. | Not determined | (51) |
| Orthoptera | *Acheta domestica* | *Citrobacter* spp. | Not determined | (52) |
| Orthoptera | *Locusta pardalina*  *Dociostaurus maroccanus*  *Calliptamus italicus*  *Chorthippus parallelus* | *Citrobacter* sp. | Not determined | (53) |

Reference

1. Burgess NRH, McDermott SN, Whiting J. Aerobic bacteria occurring in the hind-gut of the cockroach, *Blatta orientalis*. Epidemiol Infect. 1973;71(1):1–8.

2. Tatfeng YM, Usuanlele MU, Orukpe A, Digban AK, Okodua M, Oviasogie F, et al. Mechanical transmission of pathogenic organisms: The role of cockroaches. J Vector Borne Dis. 2005;42(4):129–34.

3. Akbari S, Oshaghi MA, Hashemi-Aghdam SS, Hajikhani S, Oshaghi G, Shirazi MH. Aerobic bacterial community of American cockroach Periplaneta americana, a step toward finding suitable paratransgenesis candidates. J Arthropod Borne Dis. 2015;9(1):35.

4. Chaichanawongsaroj N, Vanichayatanarak K, Pipatkullachat T, Polrojpanya M, Somkiatcharoen S. Isolation of gram-negative bacteria from cockroaches trapped from urban environment. Southeast Asian J Trop Med Public Heal. 2004;35(3):681–4.

5. Blankenchip CL, Michels DE, Braker HE, Goffredi SK. Diet breadth and exploitation of exotic plants shift the core microbiome of Cephaloleia, a group of tropical herbivorous beetles. PeerJ. 2018;6:e4793.

6. Vargas-Asensio G, Pinto-Tomas A, Rivera B, Hernandez M, Hernandez C, Soto-Montero S, et al. Uncovering the cultivable microbial diversity of costa rican beetles and its ability to break down plant cell wall components. PLoS One. 2014;9(11).

7. Hernández-García JA, Briones-Roblero CI, Rivera-Orduña FN, Zúñiga G. Revealing the gut bacteriome of *Dendroctonus* bark beetles (Curculionidae: Scolytinae): diversity, core members and co-evolutionary patterns. Sci Rep. 2017;7(1):13864.

8. Sharavati T, Chakraborti S, Modak M. Isolation and Characterization of gut bacteria from *Aspidomorpha milliaris*. World J Environ Biosci. 2012;2(1):13–20.

9. Hu X, Li M, Zhang F, Chen H. Influence of starvation on the structure of gut-associated bacterial communities in the Chinese white pine beetle (*Dendroctonus armandi*). Forests. 2016;7(6):126.

10. Hu X, Wang C, Chen H, Ma J. Differences in the structure of the gut bacteria communities in development stages of the Chinese white pine beetle (*Dendroctonus armandi*). Int J Mol Sci. 2013;14(10):21006–20.

11. Yaman M, Ertürk Ö, Aslan I. Isolation of some pathogenic bacteria from the great spruce bark beetle, *Dendroctonus micans* and its specific predator, *Rhizophagus grandis*. Folia Microbiol (Praha). 2010;55(1):35–8.

12. Hernández-García J, Gonzalez-Escobedo R, Briones-Roblero C, Cano-Ramírez C, Rivera-Orduña F, Zúñiga G. Gut Bacterial Communities of *Dendroctonus valens* and *D. mexicanus* (Curculionidae: Scolytinae): A Metagenomic Analysis across Different Geographical Locations in Mexico. Int J Mol Sci. 2018;19(9):2578.

13. Huang S, Sheng P, Zhang H. Isolation and identification of cellulolytic bacteria from the gut of *Holotrichia parallela* larvae (Coleoptera: Scarabaeidae). Int J Mol Sci. 2012;13(3):2563–77.

14. Handique G, Phukan A, Bhattacharyya B, Baruah AALH, Rahman SW, Baruah R. Characterization of cellulose degrading bacteria from the larval gut of the white grub beetle *Lepidiota mansueta* (Coleoptera: Scarabaeidae). Arch Insect Biochem Physiol. 2017;94(2):e21370.

15. Muratoglu H, Sezen K, Demirbag Z. Determination and pathogenicity of the bacterial flora associated with the spruce bark beetle, *Ips typographus* (L.)(Coleoptera: Curculionidae: Scolytinae). Turkish J Biol. 2011;35(1):9–20.

16. El-Sayed WS, Ibrahim RA. Diversity and phylogenetic analysis of endosymbiotic bacteria of the date palm root borer *Oryctes agamemnon* (Coleoptera: Scarabaeidae). BMC Microbiol. 2015;15(1):88.

17. Desai A, Bhamre P. Diversity of gut bacterial fauna of *Oryctes monocerus* linnaeus (coleoptera: scarabaeidae). Bionano Front. 2012;5:1–4.

18. Sari SLA. Cellulolytic and hemicellulolytic bacteria from the gut of *Oryctes rhinoceros* larvae. Biodiversitas, J Biol Divers. 2016;17(1):78–83.

19. Muhammad A, Fang Y, Hou Y, Shi Z. The gut entomotype of red palm weevil *Rhynchophorus ferrugineus* Olivier (Coleoptera: Dryophthoridae) and their effect on host nutrition metabolism. Front Microbiol. 2017;8:2291.

20. Tagliavia M, Messina E, Manachini B, Cappello S, Quatrini P. The gut microbiota of larvae of *Rhynchophorus ferrugineus* Oliver (Coleoptera: Curculionidae). BMC Microbiol . 2014;14(1):136.

21. Wilches DM, Laird RA, Fields PG, Coghlin P, Floate KD. Spiroplasma dominates the microbiome of khapra beetle: comparison with a congener, effects of life stage and temperature. Symbiosis. 2018;76(3):277–91.

22. Arias-Cordero E, Ping L, Reichwald K, Delb H, Platzer M, Boland W. Comparative evaluation of the gut microbiota associated with the below-and above-ground life stages (larvae and beetles) of the forest cockchafer, *Melolontha hippocastani*. PLoS One. 2012;7(12):e51557.

23. Surabhi K, Rangeshwaran R, Ml F, An S, Jagadeesh P. Isolation and characterization of the culturable microbes associated with gut of adult dung beetle *Onitis philemon* ( Fabricius ). 2018;7(2):1609–14.

24. Kuzina L V, Miller ED, Ge B, Miller TA. Transformation of Enterobacter gergoviae isolated from pink bollworm (Lepidoptera: Gelechiidae) gut with *Bacillus thuringiensis* toxin. Curr Microbiol. 2002;44(1):1–4.

25. Cheng D, Guo Z, Riegler M, Xi Z, Liang G, Xu Y. Gut symbiont enhances insecticide resistance in a significant pest, the oriental fruit fly *Bactrocera dorsalis* (Hendel). Microbiome. 2017;5(1):13.

26. Behar A, Yuval B, Jurkevitch E. Enterobacteria-mediated nitrogen fixation in natural populations of the fruit fly *Ceratitis capitata*. Mol Ecol . 2005;14(9):2637–43.

27. Behar A, Yuval B, Jurkevitch E. Gut bacterial communities in the Mediterranean fruit fly (*Ceratitis capitata*) and their impact on host longevity. J Insect Physiol. 2008;54(9):1377–83.

28. Gouveia C, Asensi MD, Zahner V, Rangel EF, de Oliveira SMP. Study on the bacterial midgut microbiota associated to different Brazilian populations of *Lutzomyia longipalpis* (Lutz & Neiva)(Diptera: Psychodidae). Neotrop Entomol. 2008;37(5):597–601.

29. Romero A, Broce A, Zurek L. Role of bacteria in the oviposition behaviour and larval development of stable flies. Med Vet Entomol . 2006;20(1):115–21.

30. Davidson EW, Rosell RC, Hendrix DL. Culturable bacteria associated with the whitefly, *Bemisia argentifolii* (Homoptera: Aleyrodidae). Florida Entomol. 2000;83(2):159.

31. Ohkuma M, Kudo T. Phylogenetic diversity of the intestinal bacterial community in the termite *Reticulitermes speratus*. Appl Environ Microbiol. 1996;62(2):461–8.

32. Upadhyaya SK, Manandhar A, Mainali H, Pokhrel AR, Rijal A, Pradhan B, et al. Isolation and characterization of cellulolytic bacteria from gut of termite. Rentech Symp Compend. 2012;1(4):14–8.

33. Thong-On A, Suzuki K, Noda S, Inoue J, Kajiwara S, Ohkuma M. Isolation and characterization of anaerobic bacteria for symbiotic recycling of uric acid nitrogen in the gut of various termites. Microbes Environ. 2009;1202010349.

34. Harazono K, Yamashita N, Shinzato N, WATANABE Y, FUKATSU T, KURANE R. Isolation and characterization of aromatics-degrading microorganisms from the gut of the lower termite *Coptotermes formosanus*. Biosci Biotechnol Biochem. 2003;67(4):889–92.

35. Adams L, Boopathy R. Isolation and characterization of enteric bacteria from the hindgut of Formosan termite. Bioresour Technol. 2005;96(14):1592–8.

36. Hayashi A, Aoyagi H, Yoshimura T, Tanaka H. Development of novel method for screening microorganisms using symbiotic association between insect (*Coptotermes formosanus* Shiraki) and intestinal microorganisms. J Biosci Bioeng. 2007;103(4):358–67.

37. Mathew GM, Lin SJ, Chang JJ, Huang CC. DGGE detection and screening of lignocellulolytic bacteria from the termite gut of *Coptotermes formosanus*. Malays J Microbiol. 2011;7:201–9.

38. French JRJ, Turner GL, Bradbury JF. Nitrogen Fixation by Bacteria from the Hindgut of Termites. J Gen Microbiol . 1976;95(2):202–6.

39. Fontes-Perez H, Olvera-García M, Chávez-Martínez A, Rodriguez-Almeida FA, Arzola-Alvarez CA, Sanchez-Flores A, et al. Genome Sequence of *Citrobacter* sp. CtB7.12, Isolated from the Gut of the Desert Subterranean Termite *Heterotermes aureus*. Genome Announc . 2015;3(6).

40. Schultz JE, Breznak JA. Heterotrophic bacteria present in hindguts of wood eating termites [*Reticulitermes flavipes* (Kollar)]. Appl Environ Microbiol. 1978;35(5):930–6.

41. Potrikus CJ, Breznak JA. Uric acid-degrading bacteria in guts of termites [*Reticulitermes flavipes* (Kollar)]. Appl Environ Microbiol. 1980;40(1):117–24.

42. Potrikus CJ, Breznak JA. Anaerobic degradation of uric acid by gut bacteria of termites. Appl Environ Microbiol. 1980;40(1):125–32.

43. Butera G, Ferraro C, Alonzo G, Colazza S, Quatrini P. The gut microbiota of the wood-feeding termite *Reticulitermes lucifugus* (Isoptera; Rhinotermitidae). Ann Microbiol. 2016;66(1):253–60.

44. Kuhnigk T, Borst E-M, Ritter A, Kämpfer P, Graf A, Hertel H, et al. Degradation of lignin monomers by the hindgut flora of xylophagous termites. Syst Appl Microbiol. 1994;17(1):76–85.

45. Sittenfeld A, Uribe-Lorío L, Mora M, Nielsen V, Arrieta G, H Janzen D. Does a polyphagous caterpillar have the same gut microbiota when feeding on different species of food plants? Rev Biol Trop. 2002;50(2):547–60.

46. Anand AAP, Vennison SJ, Sankar SG, Prabhu DIG, Vasan PT, Raghuraman T, et al. Isolation and Characterization of Bacteria from the Gut of *Bombyx mori* that Degrade Cellulose, Xylan, Pectin and Starch and Their Impact on Digestion. J Insect Sci . 2010;10(107):1–20.

47. Gandotra S, Bhuyan PM, Gogoi DK, Kumar A, Subramanian S. Screening of Nutritionally Important Gut Bacteria from the Lepidopteran Insects Through Qualitative Enzyme Assays. Proc Natl Acad Sci India Sect B Biol Sci. 2018;88(1):329–37.

48. Yaman M, Nalçacioǧlu R, Demirbaǧ Z. Studies on bacterial flora in the population of the fall webworm, *Hyphantria cunea* Drury. (Lep., Arctiidae). J Appl Entomol. 2002;126(9):470–4.

49. Brinkmann N, Martens R, Tebbe CC. Origin and diversity of metabolically active gut bacteria from laboratory-bred larvae of *Manduca sexta* (Sphingidae, Lepidoptera, Insecta). Appl Environ Microbiol. 2008;74(23):7189–96.

50. Motcha Anthony Reetha B, Mohan M. Diversity of commensal bacteria from mid-gut of pink stem borer (*Sesamia inferens* [Walker])-Lepidoptera insect populations of India. J Asia Pac Entomol . 2018;21(3):937–43.

51. Chen B, Teh B-S, Sun C, Hu S, Lu X, Boland W, et al. Biodiversity and activity of the gut microbiota across the life history of the insect herbivore *Spodoptera littoralis*. Sci Rep. 2016;6:29505.

52. Ulrich RG, Buthala DA, Klug MJ. Microbiota Associated with the Gastrointestinal Tract of the Common House Cricket, *Acheta domestica*. Appl Environ Microbiol. 1981;41(1):246–54.

53. Dillon RJ, Webster G, Weightman AJ, Dillon VM, Blanford S, Charnley AK. Composition of Acridid gut bacterial communities as revealed by 16S rRNA gene analysis. J Invertebr Pathol. 2008;97(3):265–72.

**Appendix D: Location of sampling sites**

**Table S3:** Location of sampling sites. * represents survey sites for both weevil life cycle and spatial variation.

| Site | Coordinates |
| --- | --- |
| Mamshit | 31°02'24.8"N 35°03'00.2"E |
| Dimona1 | 31°03'05.2"N 34°59'37.5"E |
| Dimona2 | 31°03'01.0"N 34°58'44.5"E |
| Yeruham | 30°58'50.8"N 34°54'04.6"E |
| Havat MaShash | 31°02'14.3"N 34°48'24.5"E |
| Neot Hovav* | 31°09'57.6"N 34°48'14.0"E |
| Mitzpe Ramon | 30°38'49.3"N 34°46'58.3"E |
| Ambassadors | 31°18'50.5"N 34°46'09.6"E |
| Tlalim | 30°59'27.5"N 34°45'43.1"E |
| Revivim | 31°03'23.9"N 34°45'14.8"E |
| Ashalim* | 30°57'28.7"N 34°40'22.2"E |

**Appendix E: Sampling and sequencing details of all analyzed samples**

**Table S4:** Summary of sequencing data, weevil species, plant species, Chao1 richness, Shannon diversity, Evenness index, and phylogenetic diversity of all analyzed samples. - represents not applicable

| Serial No. | Plant species | Weevil species | Sample type | No. of sequences | No. of OTUs | Chao1 richness | Shannon diversity | Evenness index | Phylogenetic diversity |
| --- | --- | --- | --- | --- | --- | --- | --- | --- | --- |
| 1 | *S. inermis* | *M. virgatus* | Larva | 35 132 | 1 645 | 1 282.2 | 4.8 | 0.65 | 43.8 |
| 2 | *S. inermis* | *M. virgatus* | Larva | 61 566 | 1 461 | 925.8 | 2.4 | 0.33 | 29.5 |
| 3 | *S. incanescens* | *M. virgatus* | Larva | 22 648 | 1 276 | 1 247.0 | 3.1 | 0.44 | 38.9 |
| 4 | *S. incanescens* | *M. virgatus* | Larva | 37 430 | 1 146 | 685.0 | 1.6 | 0.23 | 25.1 |
| 5 | *S. incanescens* | *M. virgatus* | Larva | 48 151 | 594 | 356.5 | 1.8 | 0.27 | 11.9 |
| 6 | *S. incanescens* | *M. virgatus* | Larva | 39 347 | 2 197 | 1 752.4 | 7.5 | 0.97 | 58.4 |
| 7 | *S. incanescens* | *M. virgatus* | Larva | 10 807 | 294 | 456.5 | 0.9 | 0.15 | 14.8 |
| 8 | *S. incanescens* | *M. virgatus* | Larva | 24 516 | 1 140 | 1 244.5 | 3.7 | 0.52 | 37.7 |
| 9 | *S. inermis* | *C. palumbus* | Larva | 32 796 | 440 | 329.6 | 1.3 | 0.22 | 10.2 |
| 10 | *S. inermis* | *C. palumbus* | Larva | 33 032 | 439 | 274.1 | 1.2 | 0.19 | 9.1 |
| 11 | *S. inermis* | *C. palumbus* | Larva | 38 626 | 851 | 661.9 | 5.3 | 0.78 | 20.8 |
| 12 | *S. inermis* | *C. palumbus* | Larva | 50 870 | 729 | 555.1 | 2.8 | 0.42 | 17 |
| 13 | *S. inermis* | *C. palumbus* | Larva | 30 498 | 410 | 357.1 | 1.4 | 0.23 | 11.3 |
| 14 | *S. inermis* | *C. palumbus* | Larva | 28 964 | 500 | 433.7 | 1.7 | 0.28 | 12.7 |
| 15 | *S. inermis* | *C. palumbus* | Larva | 32 571 | 622 | 391.0 | 2.1 | 0.33 | 15.2 |
| 16 | *S. inermis* | *C. palumbus* | Larva | 47 423 | 602 | 362.4 | 2 | 0.31 | 13.4 |
| 17 | *S. inermis* | *C. palumbus* | Larva | 26 569 | 158 | 92.5 | 0.7 | 0.14 | 3.7 |
| 18 | *S. inermis* | *C. palumbus* | Larva | 37 828 | 800 | 649.4 | 4.4 | 0.67 | 19.9 |
| 19 | *S. inermis* | *C. palumbus* | Larva | 50 006 | 583 | 390.1 | 1.9 | 0.3 | 11.9 |
| 20 | *S. inermis* | *C. palumbus* | Larva | 26 355 | 599 | 501.0 | 3.1 | 0.48 | 15.9 |
| 21 | *S. inermis* | *C. palumbus* | Larva | 19 146 | 468 | 488.0 | 2 | 0.33 | 13.8 |
| 22 | *S. inermis* | *C. palumbus* | Larva | 17 325 | 413 | 435.6 | 1.8 | 0.3 | 13.4 |
| 23 | *S. inermis* | *C. palumbus* | Larva | 18 146 | 361 | 302.2 | 1.2 | 0.21 | 10.8 |
| 24 | *S. inermis* | *C. palumbus* | Larva | 26 675 | 628 | 565.9 | 3.1 | 0.48 | 17.3 |
| 25 | *S. inermis* | *C. palumbus* | Larva | 18 800 | 328 | 225.2 | 1.2 | 0.2 | 9.5 |
| 26 | *S. inermis* | *C. palumbus* | Larva | 28 659 | 221 | 149.0 | 0.9 | 0.17 | 7 |
| 27 | *S. inermis* | *C. palumbus* | Larva | 20 343 | 413 | 312.5 | 1.5 | 0.26 | 11.7 |
| 28 | *S. inermis* | *C. palumbus* | Larva | 3 700 | 346 | 656.7 | 4.3 | 0.73 | 19.2 |
| 29 | *S. inermis* | *C. palumbus* | Larva | 43 949 | 536 | 383.0 | 1.7 | 0.28 | 12 |
| 30 | *S. inermis* | *C. palumbus* | Larva | 61 912 | 571 | 368.3 | 1 | 0.16 | 10.4 |
| 31 | *S. inermis* | *C. palumbus* | Larva | 35 699 | 463 | 310.6 | 1.3 | 0.21 | 11.2 |
| 32 | *S. inermis* | *C. palumbus* | Larva | 32 999 | 451 | 297.2 | 1.2 | 0.19 | 10.2 |
| 33 | *S. inermis* | *C. palumbus* | Larva | 38 168 | 477 | 310.2 | 1.4 | 0.23 | 10.8 |
| 34 | *S. inermis* | *C. palumbus* | Larva | 37 017 | 472 | 348.0 | 1.4 | 0.23 | 10.3 |
| 35 | *S. inermis* | *C. palumbus* | Larva | 23 750 | 340 | 313.0 | 1.2 | 0.2 | 8.2 |
| 36 | *S. inermis* | *C. palumbus* | Larva | 44 416 | 556 | 385.9 | 1.8 | 0.29 | 11.3 |
| 37 | *S. inermis* | *C. palumbus* | Larva | 35 798 | 510 | 378.2 | 1.5 | 0.24 | 11.7 |
| 38 | *S. inermis* | *C. palumbus* | Larva | 14 817 | 355 | 270.0 | 1.7 | 0.28 | 11.6 |
| 39 | *S. inermis* | *C. palumbus* | Larva | 31 286 | 409 | 322.1 | 1.2 | 0.2 | 10.6 |
| 40 | *S. inermis* | *C. palumbus* | Larva | 28 202 | 492 | 356.3 | 1.7 | 0.27 | 11.5 |
| 41 | *S. inermis* | *C. palumbus* | Larva | 17 740 | 834 | 951.2 | 2.3 | 0.34 | 30.6 |
| 42 | *S. inermis* | *C. palumbus* | Larva | 28 698 | 745 | 677.9 | 1.3 | 0.2 | 19.9 |
| 43 | *S. inermis* | *C. palumbus* | Larva | 16 896 | 954 | 1 087.6 | 3.3 | 0.49 | 35.3 |
| 44 | *S. inermis* | *C. palumbus* | Larva | 17 824 | 1 102 | 1 257.0 | 3.7 | 0.53 | 37.7 |
| 45 | *S. inermis* | *C. palumbus* | Larva | 11 587 | 400 | 426.7 | 2.4 | 0.4 | 14.1 |
| 46 | *S. inermis* | *C. palumbus* | Larva | 53 680 | 1 590 | 1 090.5 | 3.1 | 0.42 | 35.7 |
| 47 | *S. inermis* | *C. palumbus* | Larva | 12 484 | 1 449 | 1 668.0 | 7.6 | 1.04 | 56.8 |
| 48 | *S. inermis* | *C. palumbus* | Larva | 12 431 | 308 | 348.7 | 1.4 | 0.24 | 11.3 |
| 49 | *S. inermis* | *C. palumbus* | Larva | 46 336 | 1 282 | 1 027.1 | 2.6 | 0.36 | 32.8 |
| 50 | *S. inermis* | *C. palumbus* | Larva | 32 816 | 1 078 | 801.0 | 2.3 | 0.33 | 29.1 |
| 51 | *S. inermis* | *C. palumbus* | Larva | 10 075 | 1 564 | 1 823.5 | 8.9 | 1.21 | 66.7 |
| 52 | *S. inermis* | *C. palumbus* | Larva | 46 186 | 480 | 383.0 | 1.3 | 0.2 | 9.9 |
| 53 | *S. inermis* | *C. palumbus* | Larva | 55 445 | 1 588 | 1 095.2 | 3.1 | 0.42 | 35.4 |
| 54 | *S. inermis* | *C. palumbus* | Larva | 11 630 | 1 219 | 1 647.7 | 6 | 0.84 | 49.9 |
| 55 | *S. inermis* | *C. palumbus* | Larva | 18 369 | 1 275 | 1 297.6 | 5.2 | 0.72 | 43.6 |
| 56 | *S. inermis* | *C. palumbus* | Larva | 19 929 | 447 | 388.8 | 2 | 0.33 | 11.9 |
| 57 | *S. inermis* | *C. palumbus* | Larva | 57 768 | 1 473 | 1 103.8 | 2.4 | 0.33 | 31.5 |
| 58 | *S. inermis* | *C. palumbus* | Larva | 18 109 | 1 895 | 1 832.1 | 8.8 | 1.17 | 63.8 |
| 59 | *S. inermis* | *C. palumbus* | Larva | 23 100 | 228 | 207.5 | 0.6 | 0.11 | 5.6 |
| 60 | *S. inermis* | *C. palumbus* | Larva | 27 790 | 416 | 362.6 | 1.2 | 0.2 | 11.4 |
| 61 | *S. inermis* | *C. palumbus* | Larva | 15 454 | 960 | 1 171.1 | 3.1 | 0.46 | 38 |
| 62 | *S. inermis* | *C. palumbus* | Larva | 20 959 | 533 | 636.7 | 1.1 | 0.17 | 18 |
| 63 | *S. inermis* | *C. palumbus* | Larva | 39 983 | 1 953 | 1 546.7 | 4.9 | 0.64 | 48.6 |
| 64 | *S. inermis* | *C. palumbus* | Larva | 5 476 | 302 | 845.2 | 1.4 | 0.25 | 22.3 |
| 65 | *S. inermis* | *C. palumbus* | Larva | 6 944 | 299 | 558.7 | 1.2 | 0.22 | 22.3 |
| 66 | *S. incanescens* | *C. palumbus* | Larva | 19 209 | 394 | 448.0 | 1.5 | 0.25 | 11.1 |
| 67 | *S. incanescens* | *C. palumbus* | Larva | 38 965 | 638 | 551.0 | 2.3 | 0.36 | 15.4 |
| 68 | *S. incanescens* | *C. palumbus* | Larva | 33 928 | 827 | 642.3 | 5.4 | 0.8 | 20.1 |
| 69 | *S. incanescens* | *C. palumbus* | Larva | 42 651 | 500 | 378.0 | 1.4 | 0.23 | 10 |
| 70 | *S. incanescens* | *C. palumbus* | Larva | 13 031 | 1 683 | 1 861.9 | 8.8 | 1.19 | 62.4 |
| 71 | *S. incanescens* | *C. palumbus* | Larva | 35 936 | 916 | 772.5 | 1.7 | 0.25 | 24.4 |
| 72 | *S. incanescens* | *C. palumbus* | Larva | 17 071 | 932 | 1 008.7 | 3 | 0.44 | 36.2 |
| 73 | *S. incanescens* | *C. palumbus* | Larva | 68 793 | 2 222 | 1 251.4 | 4.6 | 0.6 | 44 |
| 74 | *S. incanescens* | *C. palumbus* | Larva | 43 033 | 2 431 | 2 120.0 | 8.7 | 1.12 | 62.1 |
| 75 | *S. incanescens* | *C. palumbus* | Larva | 61 960 | 2 121 | 1 390.8 | 4.7 | 0.62 | 43.3 |
| 76 | *S. incanescens* | *C. palumbus* | Larva | 19 158 | 362 | 435.0 | 0.8 | 0.13 | 11.7 |
| 77 | *S. incanescens* | *C. palumbus* | Larva | 47 083 | 1 207 | 858.8 | 2.1 | 0.29 | 27.7 |
| 78 | *S. incanescens* | *C. palumbus* | Larva | 24 492 | 2 168 | 2 053.3 | 8.7 | 1.14 | 63.7 |
| 79 | *S. incanescens* | *C. palumbus* | Larva | 49 226 | 2 086 | 1 486.7 | 5.2 | 0.68 | 48 |
| 80 | *S. incanescens* | *C. palumbus* | Larva | 38 359 | 1 027 | 801.6 | 1.7 | 0.24 | 25.9 |
| 81 | *S. incanescens* | *C. palumbus* | Larva | 29 329 | 656 | 550.0 | 1.1 | 0.16 | 15.9 |
| 82 | *S. incanescens* | *C. palumbus* | Larva | 54 302 | 1 761 | 1 077.5 | 3.6 | 0.49 | 39.5 |
| 83 | *S. incanescens* | *C. palumbus* | Larva | 19 911 | 1 110 | 1 313.3 | 2.8 | 0.41 | 36.3 |
| 84 | *S. incanescens* | *C. palumbus* | Larva | 36 606 | 1 311 | 1 241.5 | 2.7 | 0.37 | 33.1 |
| 85 | *S. incanescens* | *C. palumbus* | Larva | 17 227 | 942 | 1 196.8 | 3.1 | 0.45 | 35.2 |
| 86 | *S. incanescens* | *C. palumbus* | Larva | 22 655 | 1 204 | 1 136.1 | 3.4 | 0.48 | 37.2 |
| 87 | *S. incanescens* | *C. palumbus* | Larva | 57 252 | 1 856 | 1 383.8 | 3.7 | 0.49 | 40.2 |
| 88 | *S. incanescens* | *C. palumbus* | Larva | 19 635 | 805 | 905.9 | 2.1 | 0.31 | 29.7 |
| 89 | *S. incanescens* | *C. palumbus* | Larva | 15 414 | 1 023 | 1 305.2 | 3.7 | 0.54 | 39.1 |
| 90 | *S. incanescens* | *C. palumbus* | Larva | 31 344 | 1 758 | 1 606.6 | 4.9 | 0.66 | 49.2 |
| 91 | *S. incanescens* | *C. palumbus* | Larva | 25 166 | 1 385 | 1 208.7 | 4.2 | 0.58 | 41.3 |
| 92 | *S. incanescens* | *C. palumbus* | Larva | 14 953 | 1 180 | 1 361.9 | 4.5 | 0.64 | 45.4 |
| 93 | *S. incanescens* | *C. palumbus* | Larva | 37 537 | 2 314 | 1 800.9 | 7.2 | 0.93 | 58.4 |
| 94 | *S. incanescens* | *C. palumbus* | Larva | 17 498 | 1 833 | 2 082.4 | 8.8 | 1.17 | 63.5 |
| 95 | *S. inermis* | *C. palumbus* | Pupa | 12 875 | 622 | 779.1 | 2.1 | 0.33 | 27.2 |
| 96 | *S. inermis* | *C. palumbus* | Pupa | 10 171 | 1 260 | 1 699.8 | 5.8 | 0.81 | 54.9 |
| 97 | *S. inermis* | *C. palumbus* | Pupa | 14 562 | 1 737 | 1 890.2 | 8.7 | 1.16 | 62 |
| 98 | *S. inermis* | *C. palumbus* | Pupa | 5 558 | 751 | 1 417.6 | 4.9 | 0.74 | 43.9 |
| 99 | *S. inermis* | *C. palumbus* | Pupa | 17 724 | 1 893 | 1 832.8 | 8.7 | 1.16 | 61.9 |
| 100 | *S. inermis* | *C. palumbus* | Pupa | 15 632 | 1 781 | 1 746.6 | 8.8 | 1.18 | 64.6 |
| 101 | *S. incanescens* | *C. palumbus* | Pupa | 41 480 | 2 647 | 2 113.5 | 8.9 | 1.12 | 65.4 |
| 102 | *S. incanescens* | *C. palumbus* | Pupa | 7 377 | 1 476 | 2 183.2 | 8.6 | 1.18 | 67.4 |
| 103 | *S. incanescens* | *C. palumbus* | Pupa | 53 442 | 1 989 | 1 249.7 | 3.8 | 0.5 | 40.7 |
| 104 | *S. inermis* | *C. palumbus* | Adult | 4 708 | 477 | 1 204.3 | 5.2 | 0.84 | 31 |
| 105 | *S. inermis* | *C. palumbus* | Adult | 9 711 | 532 | 1 582.1 | 1.9 | 0.3 | 23.1 |
| 106 | *S. inermis* | *C. palumbus* | Adult | 33 264 | 830 | 1 453.6 | 5.1 | 0.76 | 51 |
| 107 | *S. inermis* | *C. palumbus* | Adult | 8 419 | 753 | 1 110.8 | 5.5 | 0.83 | 31.1 |
| 108 | *S. inermis* | *C. palumbus* | Adult | 27 877 | 1 025 | 1 236.8 | 3.8 | 0.54 | 36.8 |
| 109 | *S. incanescens* | *C. palumbus* | Adult | 11 707 | 1 202 | 1 393.8 | 4.7 | 0.66 | 49.2 |
| 110 | *S. incanescens* | *C. palumbus* | Adult | 5 487 | 1 233 | 1 998.9 | 8.8 | 1.24 | 65.8 |
| 111 | *-* | *-* | Mud chamber | 14 947 | 1 711 | 1 602.3 | 5.2 | 0.7 | 33.8 |
| 112 | *-* | *-* | Mud chamber | 21 246 | 2 671 | 2 473.2 | 8.1 | 1.03 | 53.1 |
| 113 | *-* | *-* | Mud chamber | 23 890 | 2 807 | 2 404.4 | 9.4 | 1.18 | 56.2 |
| 114 | *-* | *-* | Mud chamber | 11 246 | 1 807 | 2 092.0 | 7.6 | 1.02 | 59.9 |
| 115 | *-* | *-* | Mud chamber | 6 116 | 1 176 | 2 048.9 | 8.6 | 1.22 | 45.5 |
| 116 | *-* | *-* | Mud chamber | 70 773 | 2 692 | 1 656.0 | 8.8 | 1.12 | 45 |
| 117 | *-* | *-* | Mud chamber | 5 117 | 1 147 | 1 716.2 | 8.1 | 1.16 | 56.8 |
| 118 | *-* | *-* | Mud chamber | 68 351 | 2 929 | 1 927.2 | 9 | 1.13 | 47.8 |
| 119 | *-* | *-* | Mud chamber | 9 507 | 1 537 | 2 148.8 | 8.3 | 1.13 | 47.4 |
| 120 | *-* | *-* | Mud chamber | 9 691 | 1 671 | 2 159.9 | 9.1 | 1.22 | 49.6 |
| 121 | *-* | *-* | Mud chamber | 13 821 | 1 891 | 2 067.2 | 9.1 | 1.2 | 48.3 |
| 122 | *-* | *-* | Mud chamber | 5 464 | 783 | 1 143.7 | 7.9 | 1.18 | 33 |
| 123 | *-* | *-* | Mud chamber | 3 998 | 955 | 1 043.8 | 2.9 | 0.43 | 23.7 |
| 124 | *-* | *-* | Mud chamber | 15 067 | 2 016 | 1 987.6 | 9 | 1.18 | 46.8 |
| 125 | *-* | *-* | Mud chamber | 19 061 | 2 216 | 2 285.9 | 9.2 | 1.19 | 46.8 |
| 126 | *-* | *-* | Mud chamber | 36 520 | 2 426 | 1 849.1 | 8.9 | 1.14 | 44.8 |
| 127 | *-* | *-* | Mud chamber | 21 922 | 2 617 | 2 610.4 | 9.4 | 1.2 | 50.2 |
| 128 | *-* | *-* | Mud chamber | 13 901 | 2 082 | 2 411.8 | 9.2 | 1.21 | 47.7 |
| 129 | *-* | *-* | Mud chamber | 15 690 | 1 292 | 1 346.8 | 8.2 | 1.15 | 34.7 |
| 130 | *-* | *-* | Mud chamber | 25 004 | 2 301 | 2 063.1 | 9.3 | 1.2 | 45.9 |
| 131 | *-* | *-* | Mud chamber | 40 523 | 2 926 | 2 537.0 | 9 | 1.13 | 48.9 |
| 132 | *-* | *-* | Mud chamber | 24 414 | 1 825 | 1 532.4 | 7 | 0.94 | 37.5 |
| 133 | *-* | *-* | Mud chamber | 44 028 | 2 671 | 2 091.0 | 8.6 | 1.09 | 49.5 |
| 134 | *-* | *-* | Mud chamber | 19 768 | 1 850 | 1 715.7 | 8.9 | 1.18 | 41.4 |
| 135 | *-* | *-* | Mud chamber | 19 610 | 1 647 | 1 715.8 | 8.7 | 1.17 | 40 |
| 136 | *-* | *-* | Soil | 28 417 | 2 048 | 1 916.9 | 7.6 | 1 | 38.2 |
| 137 | *-* | *-* | Soil | 44 477 | 3 904 | 2 781.2 | 9.5 | 1.15 | 58.6 |
| 138 | *-* | *-* | Soil | 40 659 | 3 645 | 2 539.5 | 9.6 | 1.17 | 59.6 |
| 139 | *-* | *-* | Soil | 12 917 | 2 047 | 2 338.7 | 9.5 | 1.25 | 55.1 |
| 140 | *-* | *-* | Soil | 13 101 | 2 441 | 2 732.0 | 9.3 | 1.2 | 68.8 |
| 141 | *-* | *-* | Soil | 16 526 | 2 156 | 2 018.3 | 9.5 | 1.23 | 52.2 |
| 142 | *-* | *-* | Soil | 17 964 | 2 237 | 2 222.1 | 9.2 | 1.19 | 46.1 |
| 143 | *-* | *-* | Soil | 29 651 | 2 820 | 2 338.2 | 9.1 | 1.15 | 52.3 |
| 144 | *-* | *-* | Soil | 25 489 | 1 894 | 1 809.3 | 8.4 | 1.11 | 39.2 |
| 145 | *-* | *-* | Soil | 20 777 | 2 297 | 2 370.1 | 8.1 | 1.04 | 48.4 |
| 146 | *-* | *-* | Soil | 18 491 | 2 237 | 2 118.5 | 9.3 | 1.21 | 46.7 |
| 147 | *-* | *-* | Soil | 28 571 | 3 064 | 2 509.5 | 9.7 | 1.21 | 53.7 |
| 148 | *-* | *-* | Soil | 28 571 | 3 064 | 2 509.5 | 9.7 | 1.21 | 53.7 |
| 149 | *-* | *-* | Soil | 18 989 | 2 221 | 2 054.5 | 9.3 | 1.2 | 49.3 |
| 150 | *-* | *-* | Soil | 49 112 | 3 759 | 2 871.5 | 9.7 | 1.18 | 58 |
| 151 | *-* | *-* | Soil | 27 282 | 2 906 | 2 248.3 | 9.4 | 1.18 | 50.8 |
| 152 | *-* | *-* | Soil | 25 547 | 2 450 | 2 229.3 | 9.2 | 1.18 | 46 |
| 153 | *-* | *-* | Soil | 13 169 | 2 189 | 2 363.0 | 9.6 | 1.25 | 54.9 |
| 154 | *-* | *-* | Soil | 20 343 | 2 645 | 2 312.1 | 9.6 | 1.22 | 51.5 |
| 155 | *-* | *-* | Soil | 13 205 | 1 538 | 1 765.9 | 8.2 | 1.11 | 39.2 |
| 156 | *-* | *-* | Soil | 13 032 | 2 378 | 2 499.7 | 9.4 | 1.21 | 68.5 |
| 157 | *-* | *-* | Soil | 23 394 | 2 578 | 2 409.0 | 9.3 | 1.18 | 52.1 |
| 158 | *-* | *-* | Soil | 20 960 | 2 522 | 2 356.5 | 9.3 | 1.19 | 49.3 |
| 159 | *-* | *-* | Soil | 21 433 | 2 243 | 1 857.3 | 9 | 1.17 | 47.3 |
| 160 | *-* | *-* | Soil | 26 282 | 2 636 | 2 411.4 | 9.4 | 1.19 | 51.4 |
